# Alpha-glucosidase inhibitory compounds from Vietnamese lichen *Usnea baileyi*: *in vitro* and *in silico* aspects†

**DOI:** 10.1039/d4ra04449e

**Published:** 2024-10-15

**Authors:** Thanh-Hung Do, Thuc-Huy Duong, Y.-Thien Vu, Huu-Phuoc Tran, Thi-Truc-Ngan Nguyen, Jirapast Sichaem, Ngoc-Hong Nguyen, Huy Truong Nguyen, Duc-Dung Pham

**Affiliations:** a Laboratory of Biophysics, Institute for Advanced Study in Technology, Ton Duc Thang University Ho Chi Minh City 700000 Vietnam; b Faculty of Pharmacy, Ton Duc Thang University Ho Chi Minh City 700000 Vietnam nguyentruonghuy@tdtu.edu.vn; c Department of Chemistry, Ho Chi Minh City University of Education 280 An Duong Vuong Street, District 5 Ho Chi Minh City 748342 Vietnam dungpd@hcmue.edu.vn; d Research Unit in Natural Products Chemistry and Bioactivities, Faculty of Science and Technology, Thammasat University Lampang Campus Lampang 52190 Thailand; e CirTech Institute, HUTECH University 475 A Dien Bien Phu Street Binh Thanh District Ho Chi Minh City Vietnam

## Abstract

Using a bio-guided isolation on the Vietnamese lichen *Usnea baileyi* based on alpha-glucosidase inhibition, eleven compounds were isolated and structurally elucidated, namely, protocetraric acid (1), 8′-methylstictic acid (2), stictic acid (3), 4,6-diformyl-8-hydroxy-3-methoxy-1,9-dimethyl-11-oxo-11*H*-dibenzo[*b*,*e*][1,4]dioxepine-7-carboxylic acid (4), vicanicin (5), norstictic acid (6), diffractaic acid (7), barbatic acid (8), atranol (9), 5-chlorohaematommic acid (10), and eumitrin A1 (11). Their chemical structures were identified by extensive 1D and 2D NMR analysis and high-resolution mass spectroscopy and compared with those reported in literature. Protocetraric acid (1) and norstictic acid (6) were selected for further modification to derive new compounds, namely, 1a–1e and 6a. Both isolated and synthesized compounds were assessed for their alpha-glucosidase inhibitory activity. Compounds 1–6, 1a–1e, 6a, and 11 showed significant alpha-glucosidase inhibition with IC_50_ values ranging from 10.4 to 130 μM. Molecular docking was applied to the most active compounds 1–3, 6, 1a–1e, and 6a to clarify the inhibitory mechanism. Compound 1e was determined to be a mixed inhibitor through a kinetic study.

## Abstract 2

Using bio-guided isolation on the Vietnamese lichen *Usnea baileyi* based on alpha-glucosidase inhibition, eleven compounds were isolated and structurally elucidated. Compound 1e was determined to be a mixed inhibitor through a kinetic study.

## Graphical abstract

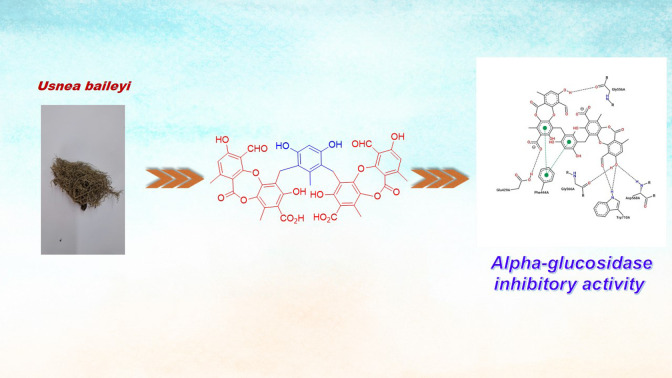

## 1. Introduction

Lichens, which are unique symbiotic systems, consist of a fungus and a photosynthetic partner. They produce a devoted chemodiversity that is endowed with various bioactivities.^1,2^ Chemical data of the fruticose lichen *Usnea baileyi* (Stirt.) Zahlbr. indicates the presence of depsides, depsidones, aliphatic and paraconic acids, dibenzofuran-related usnic acid, and xanthone dimers in them.^3,4^ Dimeric xanthones were the first significant type of compound identified within *U. baileyi*. They are yellow pigments, namely, eumitrin derivatives, as major components.^5–11^ Depsidones are believed to be the major component occurring in *U. baileyi*, comprising thirteen compounds: bailesidone, constictic acid, cryptostictic acid, hypoconstictic acid, menegazziaic acid, 8′-*O*-methylconstictic acid, methylstictic acid, 8′-*O*-methylmenegazziaic acid, 9′-*O*-methylprotocetraric acid, protocetraric acid, salazinic acid, stictic acid, and virensic acid.^6,7^ These compounds exhibit various properties such as antibacterial,^3^ antioxidant,^6^ DENV2 antiviral,^12^ and cytotoxicity against several cancer cell lines.^7^

Type 2 diabetes mellitus (T2DM) is a disease that affects the regulation of blood sugar levels in humans.^13^ In 2021, more than 536 million adults (age ranging 20–79 years) had diabetes mellitus, which is over 10.5% of the global adult population.^14^ Effective treatments for T2DM focus on controlling blood glucose levels and minimizing side effects.^15^ Alpha-glucosidase inhibitors (AGIs) are a class of oral medications that work by delaying the breakdown of complex carbohydrates into glucose, thereby reducing postprandial hyperglycemia. This mechanism of action makes them effective tools for managing T2DM.^16^ There are three well-known alpha-glucosidase inhibitors for pharmacological therapy: acarbose, miglitol, and voglibose.^16^ However, they may also cause gastrointestinal side effects, such as flatulence, diarrhea, abdominal discomfort, bloating, and nausea.^13,14^ The development of novel anti-T2DM agents has drawn extensive attention of the biochemists to reduce the limitations of current commercial drugs.^13^ Traditional medicine or natural products used in T2DM treatment have been developed due to their low toxicity and economic viability.^13,15–17^

**Fig. 1:**
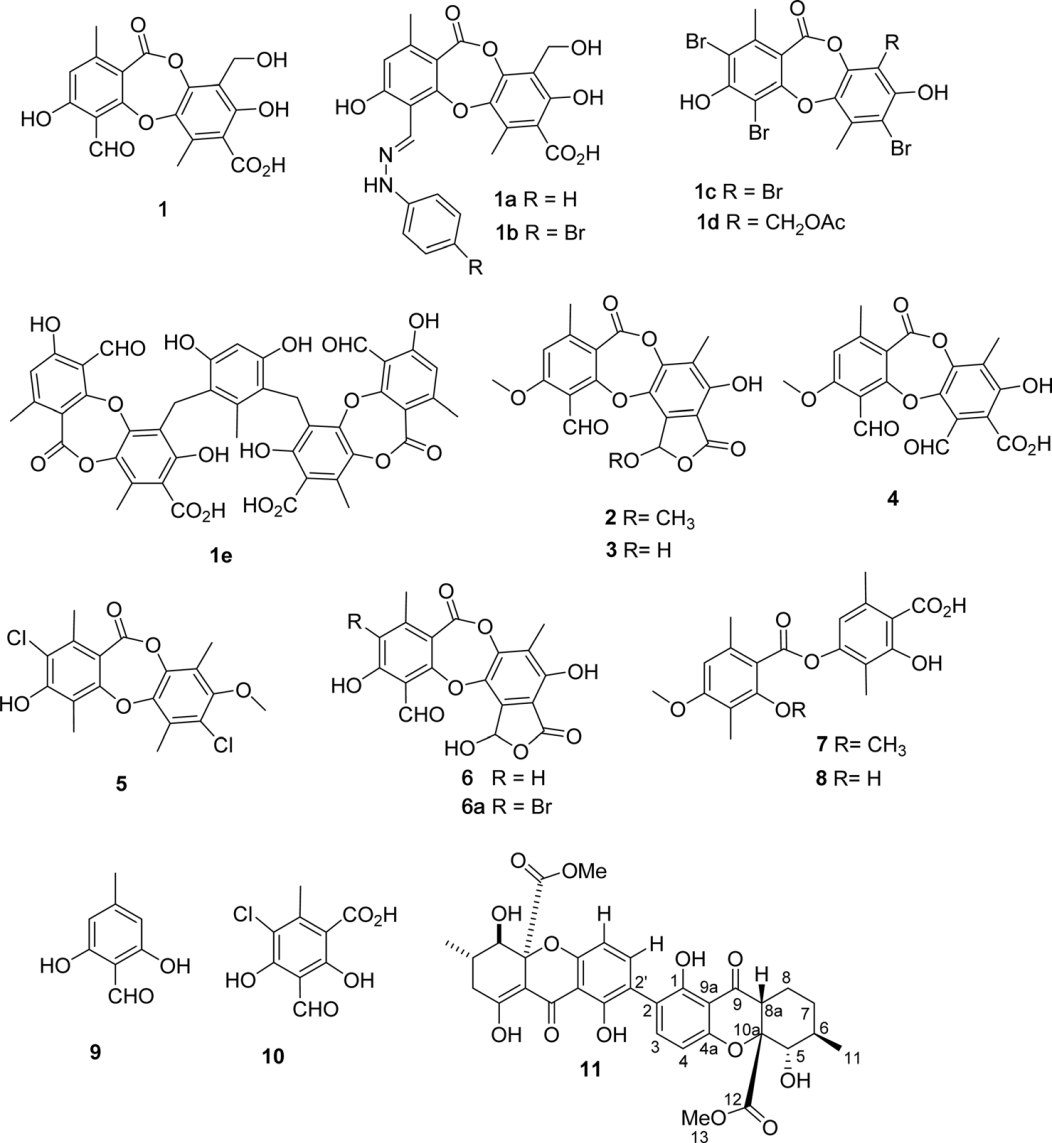
Chemical structures of 1–11, 1a–1e, and 6a.

**Fig. 2:**
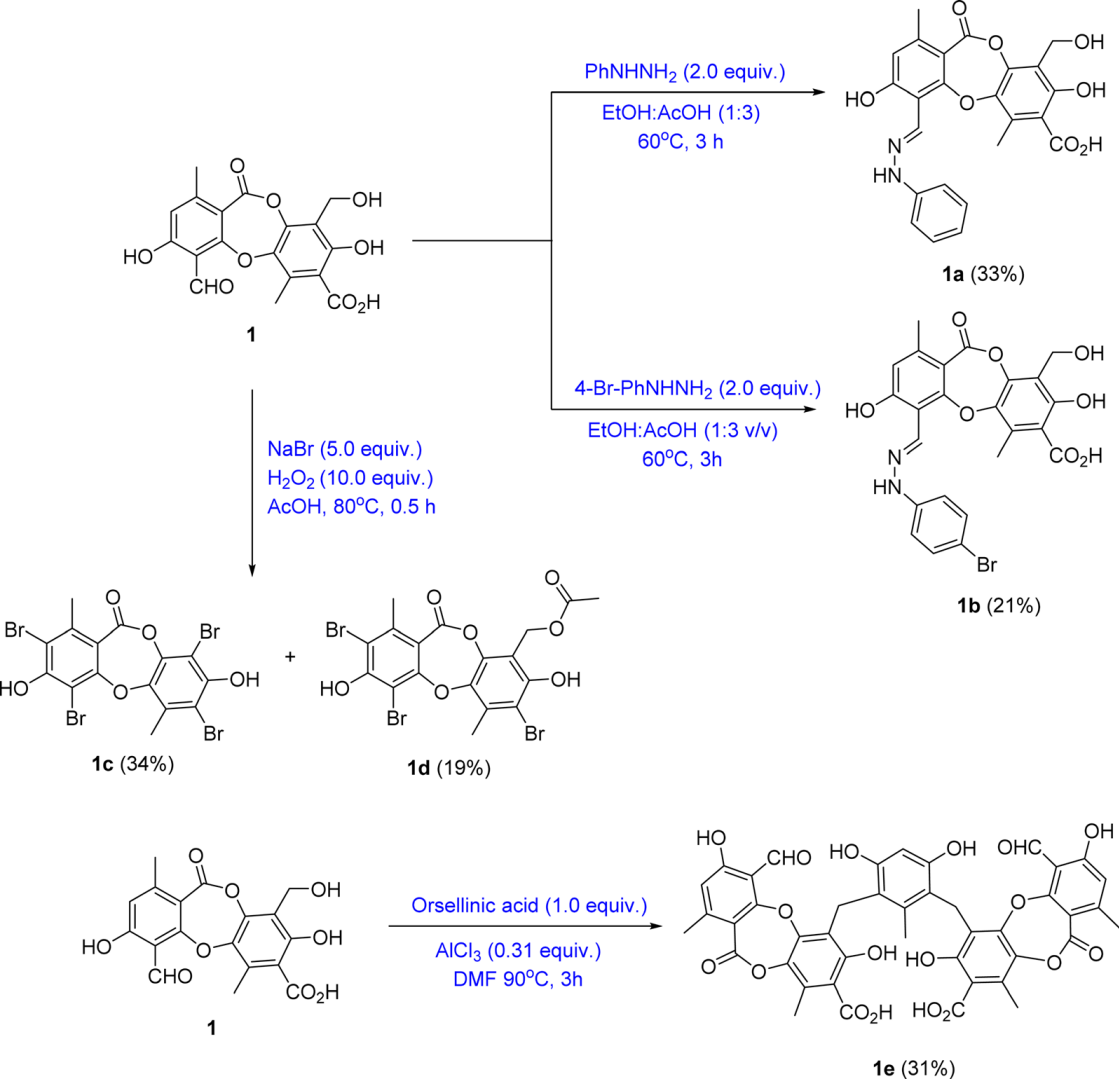
General reaction procedure from 1 to 1a–1e.

Among the depsidones mentioned above, salazinic acid and protocetraric acid are thought to be potent alpha-glucosidase inhibitors.^9,18^ Little is known about the alpha-glucosidase inhibitory activity of extracts of *U. baileyi* and their constituents. Recently, a few eumitrin derivatives were reported as moderate alpha-glucosidase inhibitory compounds.^10^ In the search for new alpha-glucosidase inhibitors from lichen sources, a bioactive-guided investigation was performed on the Vietnamese *U. baileyi*. Eleven compounds were isolated and structurally elucidated: protocetraric acid (1),^9^ 8′-methylstictic acid (2),^19^ stictic acid (3),^20^ 4,6-diformyl-8-hydroxy-3-methoxy-1,9-dimethyl-11-oxo-11*H*-dibenzo[*b*,*e*][1,4]dioxepine-7-carboxylic acid (4),^21^ vicanicin (5),^22^ norstictic acid (6),^23^ diffractaic acid (7),^24^ barbatic acid (8),^25^ atranol (9),^26^ 5-chlorohaematommic acid (10),^27^ and eumitrin A1 (11)^28^ (Fig. 1). Protocetraric acid (1) and norstictic acid (6) were further transformed into new derivatives 1a–1e and 6a using several procedures (Fig. 1 and 2). Their structures were elucidated by extensive spectroscopic analysis (HRESIMS and NMR) and compared with those reported in literature. Isolated and synthesized compounds were evaluated for their alpha-glucosidase inhibition. Molecular docking studies of selected compounds were also conducted to discover the inhibitory mechanism of the compounds.

## 2. Results and discussion

**Scheme 1:**
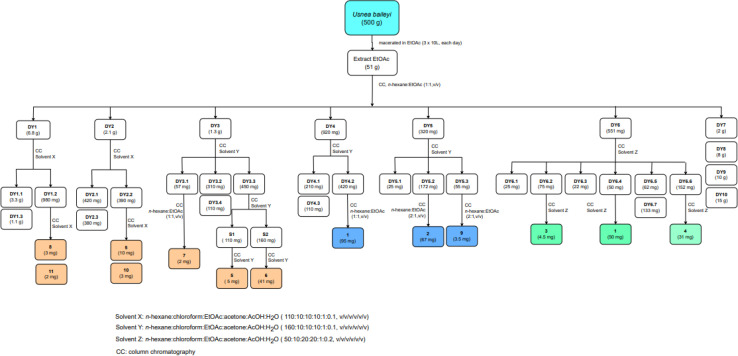
The isolation procedure of 1–11.

Bioactive-guided isolation of the Vietnamese *U. baileyi* was conducted based on alpha-glucosidase inhibition. The lichen material was macerated in *n*-hexane, EtOAc, and MeOH, providing corresponding extracts. These extracts were screened for activity, with the EtOAc extract being the most active extract with an IC_50_ value of 34.5 ± 1.2 μg mL^−1^ (Table S1†). Fractions DY1-DY10 were prepared from the EtOAc extract using silica gel column chromatography (Scheme 1) and then evaluated for alpha-glucosidase inhibitory activity (Table S1†). Fractions DY1–DY5 were selected for further chemical analysis. The results in the isolation of eleven compounds included protocetraric acid (1), 8′-methylstictic acid (2), stictic acid (3), 4,6-diformyl-8-hydroxy-3-methoxy-1,9-dimethyl-11-oxo-11*H*-dibenzo[*b*,*e*][1,4]dioxepine-7-carboxylic acid (4), vicanicin (5), norstictic acid (6), diffractaic acid (7), barbatic acid (8), atranol (9), 5-chlorohaematommic acid (10), and eumitrin A1 (11). Compounds 4, 5, and 10 were first reported in *U. baileyi*. The isolated compounds were classified into four classes: depsidones (1–6), depsides (7 and 8), monoaromatic compounds (9 and 10), and dimeric xanthone (11).

**Fig. 3:**
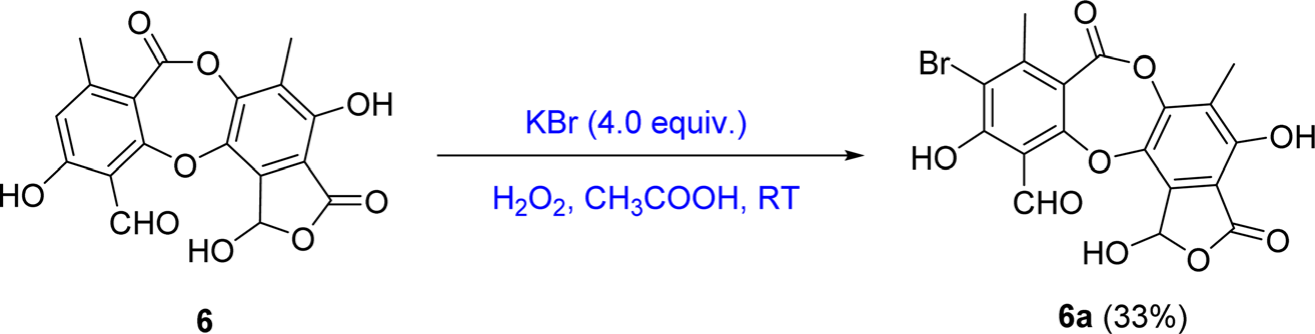
General reaction procedure from 6 to 6a.

Based on the larger amounts of 1 and 6, they were selected for synthetic modification (Fig. 2 and 3). The imine formation and aromatic bromination were consecutively applied to 1 to obtain derivatives 1a–1d. These reactions were selected based on the enhancement of the biological activity of similar derivatives of salazinic acid.^18^ Bromination was conducted on 6, giving the 4-Br-substituted product 6a (Fig. 3). Isolated yields are shown in Fig. 2 and 3. NMR data of 1a–1d are presented in Tables 1 and 2. The mechanism from 1 to degraded products 1c and 1d was previously reported.^18^ Compound 1 was also subjected to Friedel–Crafts alkylation with orsellinic acid to form 1e. The conditions followed those of a previous report with modifications.^18^ The NMR comparison of 1e and parmosidone K^29^ was highly similar, indicating that 1e was a parmosidone-A-type *meta*-depsidone. The downfield methyl at *δ* 2.61 is indicative of this skeleton.^29^ The chemical structure 1e was further supported by a combination of 1D and 2D NMR and HRESI mass spectra. Nguyen and co-workers reported the transformation from a *para*-depsidone 1 to a *meta*-depsidone 1e.^9^ Initially, compound 1 was transformed to parmosidone A *via* a key Smile rearrangement (Fig. S8†).^9^ Simultaneously, orsellinic acid was decarboxylated to form orcinol (Fig. S8†). Next, two consecutive alkylations between two parmosidone-A units and orcinol were activated to form 1e. The last transformation was similar to the formation of parmosidone F from salazinic acid.^18^ This reaction was selected due to the dramatic enhancement of the activity of parmosidone F compared to salazinic acid.^18^

**Table 1:** ^1^H (500 MHz) NMR data of 1 and 1a–1d in acetone-*d*_6_

No.	1	1a	1b	1c	1d
	*δ*_H_, mult (*J* in)	*δ*_H_, mult (*J* in)	*δ*_H_, mult (*J* in)	*δ*_H_, mult (*J* in)	*δ*_H_, mult (*J* in)
5	6.83 (s)	6.76 (s)	6.71 (s)		
8	10.59 (s)	8.90 (s)	8.86 (s)		
9	2.43 (s)	2.50 (s)	2.42 (s)	2.50 (s)	2.53 (s)
8′	4.60 (s)	4.78 (s)	4.79 (s)		5.27 (s)
9′	2.40 (s)	2.72 (s)	2.67 (s)	2.72 (s)	2.73 (s)
11′					1.99 (s)
2′′/6′′		7.03 (d, 7.5)	6.96 (d, 7.0)		
3′′/5′′		7.32 (t, 7.5)	7.42 (d, 7.4)		
4′′		6.90 (t, 7.5)			
2′-OH			10.74 (brs)		
8′-OH		3.57 (br)	4.13 (br)		
NH-1′′		10.78 (s)	10.31 (s)		

**Table 2:** ^13^C (125 MHz) NMR data of 1 and 1a–1d in acetone-*d*_6_

No.	1	1a	1b	1c	1d
	*δ*_C_	*δ*_C_	*δ*_C_	*δ*_C_	*δ*_C_
1	112.4	113.9	112.4	110.6	109.2
2	161.1	161.9	159.6	156.9	156.6
3	111.8	111.8	109.7	100.8	100.6
4	163.8	162.5	162.4	159.7	159.8
5	117.0	117.2	117.3	113.2	114.8
6	152.0	145.8	144.1	142.8	143.4
7	163.9	161.1	161.1	161.1	161.1
8	191.7	136.0	136.9		
9	21.3	21.3	21.4	22.4	22.4
1′	116.6	110.0	112.2	101.1	101.2
2′	154.9	154.5	154.5	150.6	151.8
3′	118.6	118.1	119.6	113.7	122.6
4′	144.5	146.9	147.2	143.3	143.6
5′	141.7			144.2	144.3
6′	129.4			131.6	132.9
7′	170.1	173.1	173.1		
8′	52.9	54.3	54.2		57.4
9′	14.3	15.5	16.5	19.6	19.6
10′					171.9
11′					20.7
1′′		145.8	143.6		
2′′/6′′		113.0	114.8		
3′/5′′		130.4	133.1		

Compounds 1–11, 1a–1e, and 6a were evaluated for their alpha-glucosidase inhibition (Table 3). Compounds 1–3, 6, and 11 exhibited good activity, with IC_50_ values ranging from 30.4 to 130 μM. Compounds 4, 5, and 7–10 showed weak activity. Generally, synthetic compounds 1a–1e and 6a showed potent activity against alpha-glucosidase, with IC_50_ values ranging from 10.4 to 102.0 μM, stronger than the reference compound acarbose.

**Table 3:** Alpha-glucosidase inhibition of compounds 1–11, 1a–1e, and 6a

Compound	IC_50_ (μM)
1	70.6 ± 1.2
1a	25.9 ± 2.2
1b	20.7 ± 1.1
1c	80.7 ± 1.8
1d	91.8 ± 1.5
1e	10.4 ± 0.4
2	41.8 ± 1.5
3	30.4 ± 1.4
4	>200
5	>200
6	50.7 ± 2.7
6a	102 ± 1.8
7	>200
8	>200
9	>200
10	>200
11	130 ± 2.0
Acarbose	361 ± 2.2

The inhibitory order was ranked as follows: depsidones (1–6, 1a–1e, and 6a) > xanthone (11) > depsides (7 and 8)/monoaromatic compounds (9 and 10). The alpha-glucosidase inhibition of protocetraric acid (1), diffractaic acid (7), and atranol (9) have been previously reported.^9,11^ Particularly, protocetraric acid was reported as a moderate inhibitor with an IC_50_ value of 81.6 μM. In contrast, diffractaic acid (7) and atranol (9) showed weak activity, with IC_50_ values of 419.6 and 559.2 μM, respectively. These data are consistent with the current ones shown in Table 3. The dimeric xanthone eumitrin A1 (11) showed moderate activity, consistent with those of eumitrins I–K.^10^ Monoaromatic compounds 9 and 10 exhibited weak inhibition. Compared to the lichen-derived monoaromatic compounds,^30^ the presence of a substituent at C-3 might affect the activity. A comparison of the activities of the natural depsidones 1–6 indicated that 3 was the strongest inhibitor. This finding proposed that the occurrence of the 8′-methoxy (in 2) and 4-methoxy groups (in 6) or the disappearance of a *γ*-lactone moiety (in 1, 4, and 5) dramatically decreased the activity.

**Fig. 4:**
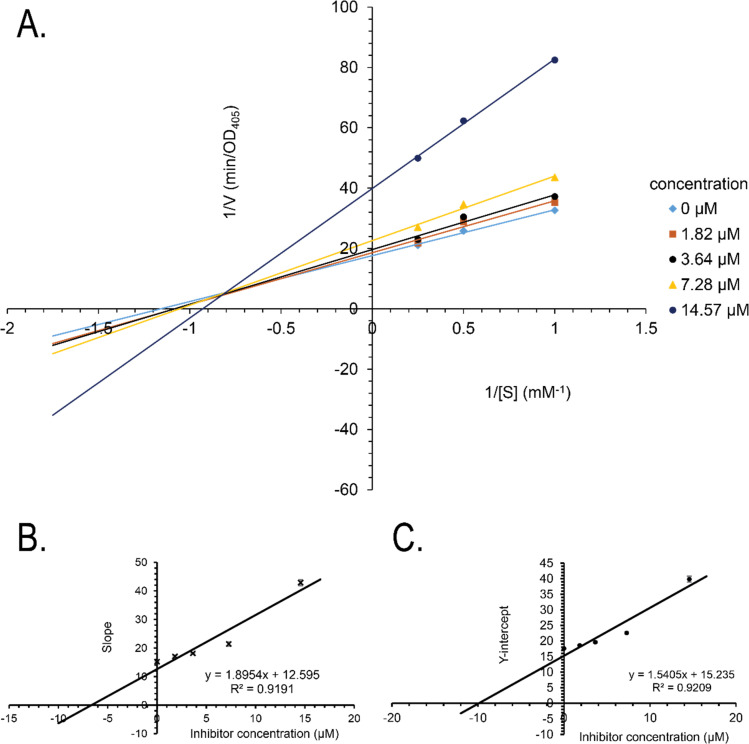
(A) Lineweaver–Burk plot for alpha-glucosidase inhibition by 1e, (B) secondary plot of the slope *vs.* inhibitor concentration, and (C) secondary plot of the *Y*-intercept *vs.* inhibitor concentration.

Synthetic compounds 1a, 1b, and 1e are much stronger than the original compound 1 (IC_50_ 70.6 μM), while 1c and 1d are weaker. Replacing the imine group in 1a and 1b for the aldehyde group in 1 enhances the activity. Notably, the synthetic compound 1e is seven-fold stronger than 1, highlighting the significant role of the orcinol unit in 1e. In contrast, the bromine atoms within a depsidone skeleton (1c, **1d**, and 6a) decrease the activity. Compound 1e showed the strongest alpha-glucosidase inhibition; thus, it was further selected to investigate the inhibition mechanism. At concentrations of 0, 1.82, 3.64, 7.28, and 14.57 μM of 1e, the activity was evaluated. Lineweaver–Burk plots gave an intersection of different lines within the second quadrant (Fig. 4), indicating that 1e acted as a mixed-mode inhibitor. This type was identical to that of parmosidone F.^18^ An imine product derived from salazinic acid, namely, (*E*)-11-((2-(4-bromophenyl)hydrazineylidene)methyl)-1,4,10-trihydroxy-5-(hydroxymethyl)-8-methyl-7*H*-benzo[6,7][1,4]dioxepino[2,3-*e*]isobenzofuran-3,7(1*H*)-dione^18^ and *meta*-depsidone cristifone B are defined as non-competitive inhibitors.^30^ The inhibition constants of 1e binding with the free enzyme (*K*_i_) and with the enzyme–substrate complex 
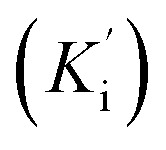
 were determined to be 6.65 ± 0.48 μM and 9.89 ± 0.47 μM, respectively (Fig. 4B and C). The value of *K*_i_ was lower than that of 
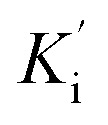
, indicating that the binding affinity of alpha-glucosidase-1e exceeded that of alpha-glucosidase-PNPG-1e complex.

Depsidones 1–6, 1a–1e, and 6a and bixanthone 11 were evaluated for cytotoxicity against normal cells Hek293 and liver cancer cell line HepG2. The results are shown in Table S2.† All depsidones are inactive (IC_50_ > 100 μM), except for 1. These IC_50_ values indicated that synthesized compounds 1a–1e were less cytotoxic than their starting material. In contrast, compound 11 showed moderate activity toward both cell lines, with IC_50_ values of 60.3 μM (HeK293) and 62.1 μM (HepG2).

**Fig. 5:**
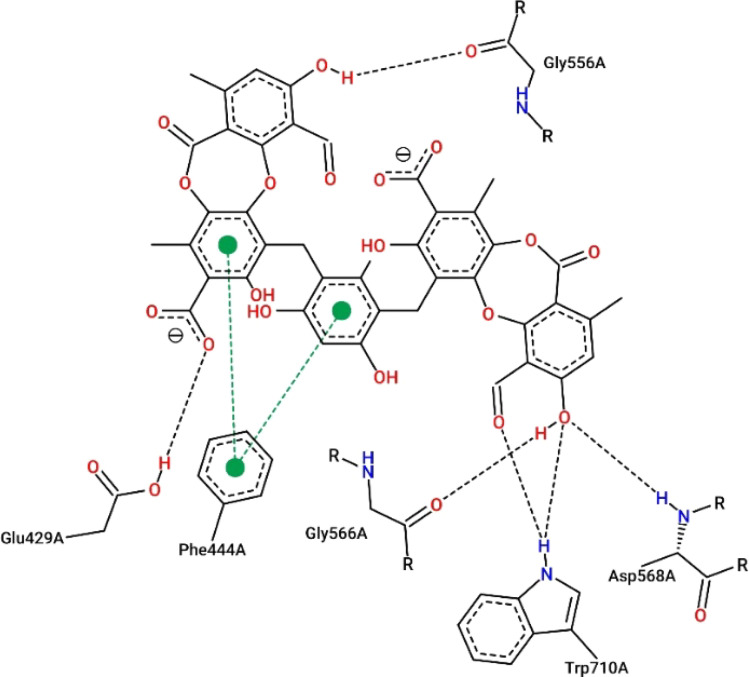
Interactions between compound 1e and the residues within the binding pocket. The black and green dashed lines represent hydrogen bonds and π–π stacking, respectively.

**Fig. 6:**
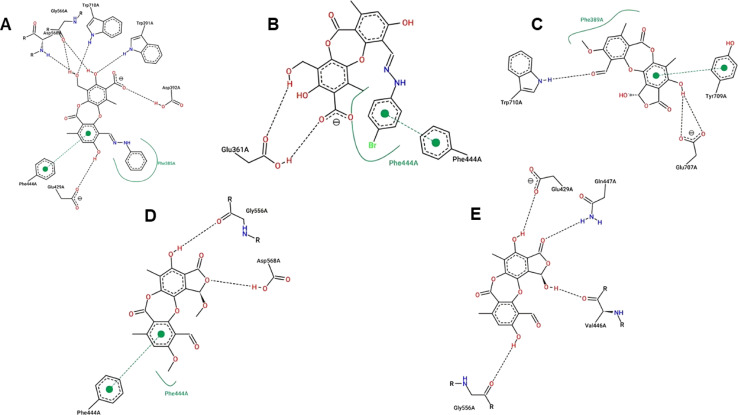
Interactions between compounds 1a (A), 1b (B), 2 (D), 3 (C), and 6 (E) and the residues within the binding pocket. The black and green dashed lines and the green curves represent hydrogen bonds, π–π stacking, and hydrophobic interactions, respectively.

Virtual screening investigations were performed for compounds 1, 1a–1e, 2, 3, 6, and 6a with acarbose as a reference to clarify their alpha-glucosidase inhibitor potential (Fig. 5 and 6). The crystal structure of alpha-glucosidase I (PDB ID: 4 J5T) was utilized for molecular docking of the synthesized compounds. AutoDock Vina was employed to evaluate the binding affinity of these complexes. Experimental and docked free energy of binding are presented in Table 4, with interactions of 1e and the binding pocket of alpha-glucosidase illustrated in Fig. 5. Notably, all tested compounds exhibited stronger inhibitory activity against alpha-glucosidase compared to acarbose. A substantial correlation (*R* = 0.81) was observed between experimental IC_50_ values and docking results, indicating the reliability of *in silico* screening in evaluating alpha-glucosidase activity across the synthesized compounds.

**Table 4:** Experimental alpha-glucosidase inhibition and predicted energy of binding^a^

Ligand	IC_50_ (μM)	Δ*G*_EXP_ (kcal mol^−1^)	Δ*G*_DOCK_ (kcal mol^−1^)
1	70.6 ± 1.2	−5.57	−9.3
1a	25.9 ± 2.2	−6.25	−9.5
1b	20.7 ± 1.1	−6.39	−9.6
1c	80.7 ± 1.8	−5.58	−8.2
1d	91.8 ± 1.5	−5.50	−9.1
1e	10.4 ± 0.4	−6.79	−11.2
2	41.8 ± 1.5	−5.97	−8.8
3	30.4 ± 1.4	−6.16	−8.8
6	50.7 ± 2.7	−5.86	−9.2
6a	102 ± 1.8	−5.44	−8.2
Acarbose	361 ± 2.2	−5.17	−8.4

a The experimental free energy (Δ*G*_EXP_) of the binding of molecules was estimated from the IC_50_ values: 
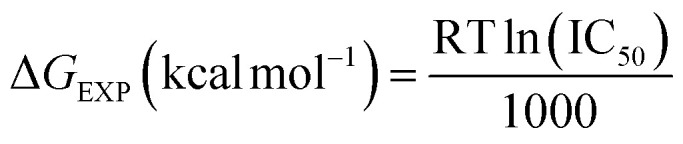
. IC_50_ value was assumed to be equal to the inhibition constant (*K*_i_).

Compound 1e proved to be a promising alpha-glucosidase inhibitor, demonstrating the lowest IC_50_ value (10.4 μM) and a corresponding docking score of −11.2 kcal mol^−1^. The results of molecular docking highlighted the interactions between 1e and critical amino acid residues within the enzyme's binding pocket, encompassing Glu^429^, Phe^444^, Gly^566^, Asp^568^, and Trp^710^.^31^ The bulky and unique structural attributes of 1e, mainly its structure composed of two protocetraric acids connected *via* an orcinol bridge, engaged in π–π stacking interactions with Phe^444^ and established multiple hydrogen bonds with adjacent residues. Therefore, compound 1e effectively occupied the enzyme's binding pocket and formed substantial interactions, resulting in potent inhibition of alpha-glucosidase activity.

Compounds 1a and 1b, derivatives of imination reactions with phenylhydrazine derivatives exhibited similar binding interactions within the enzyme's binding pocket. In particular, 1a was able to form hydrogen bonds with Trp^391^, Asp^392^, Glu^429^, Gly^566^, Asp^568^, and Trp^710^ (Fig. 6). Additionally, the presence of the phenyl ring of protocetraric acid enabled π–π interactions with Phe^444^. At the same time, the phenylhydrazinyl moiety fostered hydrophobic interactions with Phe^385^, contributing to the moderate binding affinity and IC_50_ (−6.25 kcal mol^−1^ and 25.9 μM, respectively). Moreover, the additional bromide at the *para* position of the phenylhydrazinyl moiety further enhanced hydrophobic interactions and π–π stacking with Phe^444^, resulting in a lowered IC_50_ value (20.7 μM).

In contrast, compounds 2, 3, and 6 shared a similar scaffold characterized by a cyclization reaction involving the carboxyl group, leading to the incorporation of a furanone ring into the protocetraric acid scaffold. In spite of this structural modification, the presence of the condensed furanone ring did not effectively increase the inhibitory capacity of these compounds compared to 1a and 1b (Fig. 6). This observation is supported by the comparable IC_50_ values (41.8, 30.4, and 50.7 μM, respectively) and corresponding docking scores for compounds 2, 3, and 6 (−5.97, −6.16, and −5.86 kcal mol^−1^, respectively).

An appropriate correlation coefficient of *R* = 0.81 was found between the experimental binding affinities and the docking results, suggesting that AutoDock 4.2 effectively evaluated the molecular mechanism underlying the action of the ligands on the alpha-glucosidase protein. Interactions between the protein and ligands were analyzed using MOE 2015.10. Hydrophobic contacts have been confirmed to play an essential role in the binding affinity of protein-ligand complexes.

**Fig. 7:**
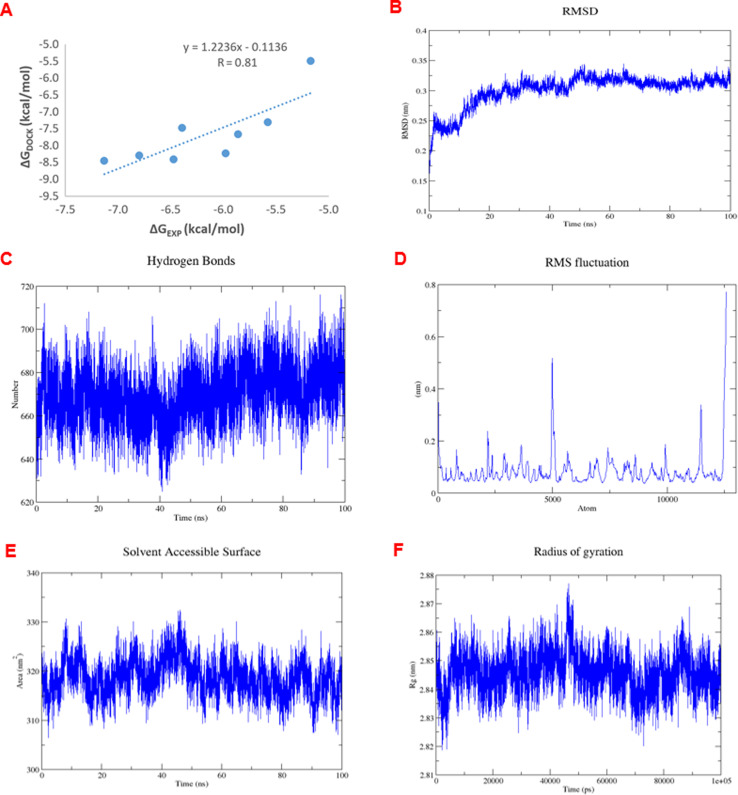
(A) The correlation between experimental and docked free energies of binding, *R* = 0.81. (B) RMSD of complex 1e-4J5T as a function of computed time. (C) Variations in the number of hydrogen bonds in the 1e-4J5T complex over simulation time. (D) RMSF of the C-alpha residue as a function of residue number. (E) Variations in the area of solvent accessible surface of 1e-4J5T complex over simulation time. (F) Variations in the radius of gyration of 1e-4J5T complex over simulation time.

The analysis of RMSDs of 1e in Fig. 7A–F shows that the fluctuations of the whole system vary less than 3 Å (0.15–0.35 nm) over the 100 ns period and only 1 Å (0.25–0.35 nm) after 50 ns, which indicates a suitably stable and equilibrated complex. The insignificant changes in the hydrogen bonds (Fig. 7C), solvent-accessible surface area (Fig. 7E), and radius of gyration (Fig. 7E) of the complex as a function of time further suggested stable complex formation. Furthermore, the RMSF values indicating the level of movement of C-alpha residues (Fig. 7D) alter chiefly under 0.3 nm, thus confirming the stability in the coordinates of the complex over time. Therefore, we designated the simulation data in the 50–100 ns range to compute the free binding energy by the MM/PBSA approach over 50 frames. The obtained values are shown in Table 5.

**Table 5:** Free binding energy determined by MMPBSA method

Unit	Δ*E*_elec._	Δ*E*_vdW_	Δ*G*_sa_	Δ*G*_polar_	Free binding energy
kJ mol^−1^	−7.52	−28.96	−4.21	24.30	−16.39

**Fig. 8:**
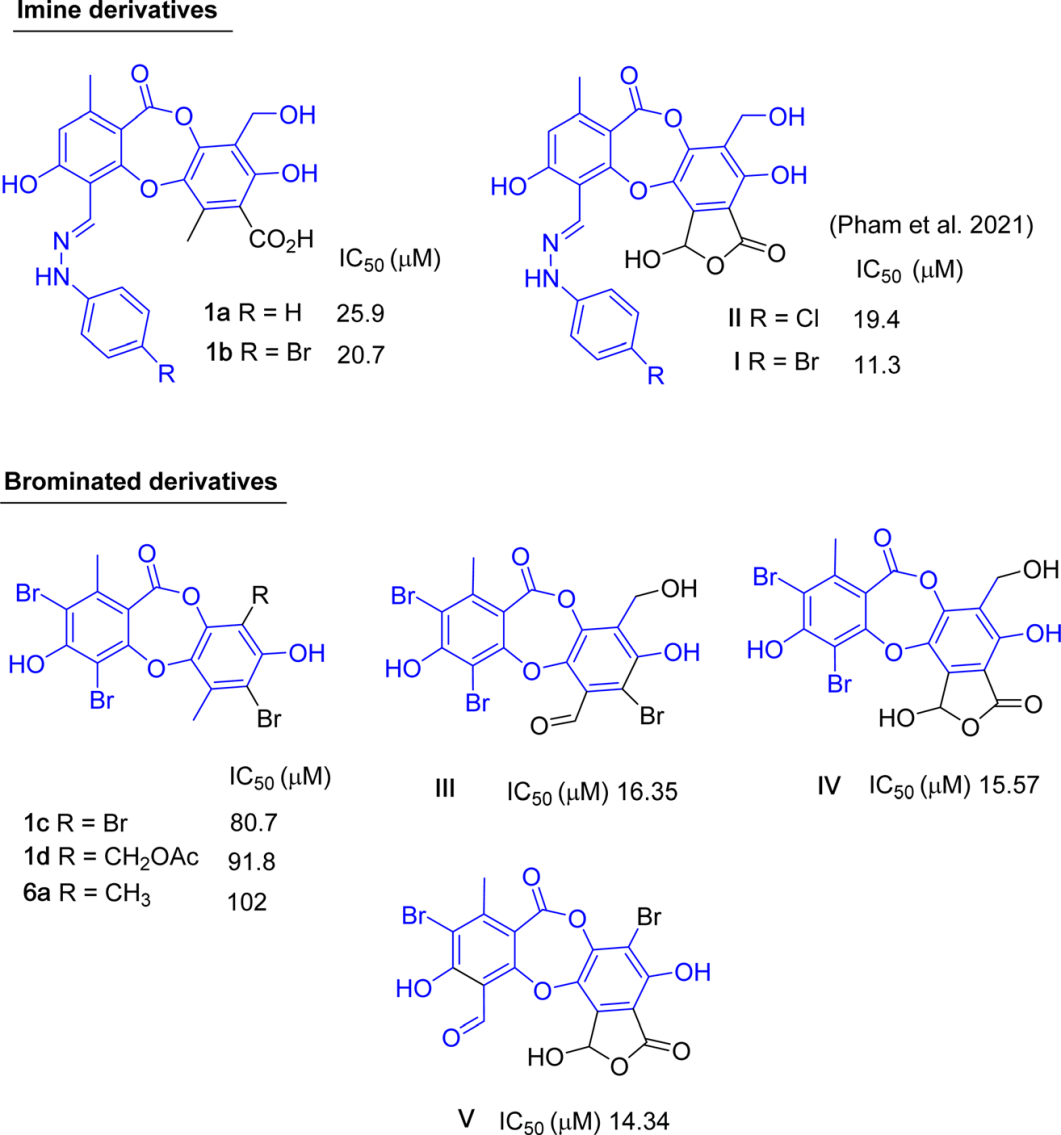
Chemical structures of 1a–1d, 6a, and related analogs, along with their IC_50_ values of alpha-glucosidase inhibition.

**Fig. 9:**
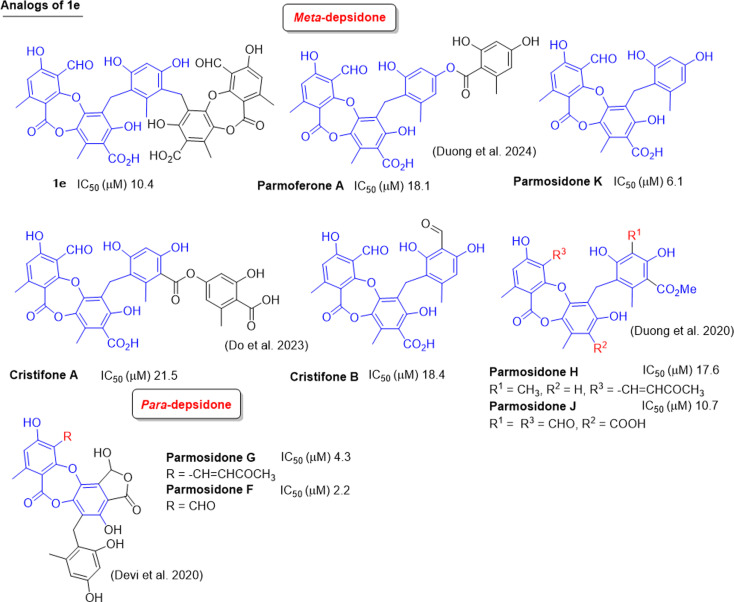
Natural analogs of 1e and their IC_50_ values of alpha-glucosidase inhibition.

Up to now, dozens of natural lichen-derived depsidones that inhibit alpha-glucosidase have been reported.^29,30,32,33^ Over 30 synthetic analogs have been prepared using salazinic acid and protocetraric acid as starting materials.^9,18^ The relationship between the structure and activity of synthetic products 1a–1e and 6a and previously related analogs was reviewed, as shown in Fig. 8 and 9. There are three series: imine-based, bromination-based, and tricyclic-scaffold depsidones. Regarding imine derivatives 1a, 1b, I, and II (Fig. 8), the γ-lactone ring in I and II significantly increased the activity. It is noted that brominated derivatives 1c, 1d, 6a, and III–V shared the same depsidone scaffold (highlighted in blue in Fig. 8). The stronger activity of III–V compared to 1c, 1d, and 6a might come from the different substituents in the B-ring. Interestingly, the γ-lactone ring in IV and V plays an important role in the inhibition.

Two types of tricyclic scaffold depsidones are depicted in Fig. 9: *meta*- and *para*-depsidones. *Para*-depsidones, such as parmosidones G and H, are significantly stronger than *meta*-depsidones, indicating the importance of the skeleton type. Among *meta*-depsidones, parmosidone K is believed to be the most active compound. Considering the structural features, other compounds are considered derivatives of parmosidone K, which has additional moieties. As seen in Fig. 9, various substituents in the C-ring decrease the activity. In parmosidones H and J, the transformation of the 3-CHO group gives a negative effect toward alpha-glucosidase inhibition.

## 3. Materials and methods

### 3.1. Source of the lichen material

The lichen *Usnea baileyi* was collected from tree bark in Duc Trong, Lam Dong province, Vietnam, in May 2023. The voucher specimen was deposited at University of Education, Ho Chi Minh City, Vietnam (registration No. UE-L012). The sample was identical with that of a previous report.^7^

### 3.2. Extraction and isolation of 1–11 from the lichen

A dry ground weight of 500 g was extracted with EtOAc (3 × 10 L, each day) at room temperature. The combined EtOAc solutions were concentrated under reduced pressure to yield 51 g of residue. The EtOAc extract was dissolved in acetone to obtain a solid (4 g) and an acetone-dissolved solution. The obtained solution was applied to silica gel column chromatography and eluted with *n*-hexane:EtOAc (1 : 1, v/v) to provide ten fractions, DY1–DY10. The isolation procedure with details of compounds 1–11 is shown in Scheme 1.

### 3.3. Synthetic procedures

Protocetraric acid (1) (20.0 mg, 0.053 mmol) and phenylhydrazine (23.1 mg, 0.214 mmol) were dissolved in a mixture of 1.5 mL acetic acid and 0.5 mL ethanol. The solution was stirred at 60 °C for 3 hours. After cooling, the reaction mixture was extracted with EtOAc : H_2_O (1 : 1, v/v). The organic layer was evaporated under reduced pressure to obtain a crude yellow product. The crude product was applied to silica gel column chromatography and eluted with a gradient of chloroform : EtOAc : acetone : acetic acid (100 : 40 : 24 : 8, v/v/v/v) to obtain product 1a (8 mg, 33%).

Compound 1 (20.0 mg, 0.053 mmol) and 4-bromophenylhydrazine hydrochloride (48.0 mg, 0.214 mmol) were dissolved in a mixture of 1.5 mL acetic acid and 0.5 mL ethanol. The solution was stirred at 60 °C for 3 hours. After cooling, the reaction mixture was extracted with EtOAc : H_2_O (1 : 1, v/v). The organic layer was evaporated under reduced pressure to obtain a crude yellow product. The crude product was applied to silica gel column chromatography and eluted with a gradient of chloroform : EtOAc : acetone : acetic acid (100 : 40 : 24 : 8, v/v/v/v) to obtain 1b (6 mg, 21%).

In 2.0 mL of a mixture of acetic acid, 1 (40.0 mg, 0.107 mmol) and sodium bromide (55.0 mg, 0.535 mmol) were dissolved at 80 °C. 0.1 mL (1.07 mmol) of 30% hydrogen peroxide (0.18 mmol) was added to the reaction mixture. The reaction was conducted for 30 minutes. The solution was neutralized with saturated sodium hydrogen carbonate and then extracted with ethyl acetate-water (1 : 1, v/v) to gain an organic layer. This layer was subsequently washed with brine three times, dried and applied to silica gel CC, and eluted with a gradient of *n*-hexane : EtOAc : acetone (7 : 1 : 0.01, v/v/v) to obtain 1c (21.4 mg, 34%) and 1d (11.6 mg, 19%).

A solution of 1 (100.0 mg, 0.267 mmol) and orsellinic acid (44.9 mg, 0.267 mmol) in dimethylformamide (2.0 mL) was added to AlCl_3_ (11 mg, 0.0824 mmol). The mixture was stirred at 90 °C for 3 hours. The resulting solution was extracted with ethyl acetate:water (1 : 1, v/v) to gain an organic layer. This layer was subsequently washed with brine three times, dried and applied to silica gel CC, and eluted with a gradient of chloroform : EtOAc : acetone : acetic acid (100 : 40 : 24 : 8, v/v/v/v) to obtain 1e (69.2 mg, 31%).

The mixture of 6 (20 mg, 0.054 mmol) and potassium bromide (5.4 mg, 0.045 mmol) was dissolved in 0.4 mL mixture of acetic acid. A solution of hydrogen peroxide 30% (0.04 mL, 0.5 mmol) was added, and the solution was stirred at room temperature for 60 minutes. After cooling, the reaction mixture was neutralized with sodium bicarbonate and extracted with EtOAc : H_2_O (1 : 1, v/v). The organic layer was evaporated under reduced pressure to obtain crude product. The crude product was applied to silica gel column chromatography and eluted with a gradient of *n*-hexane : chloroform : EtOAc : acetone : acetic acid (80 : 40 : 8 : 5 : 2, v/v/v/v/v) to obtain product 6a (6.7 mg, 33%).

(1a) (*E*)-3,8-dihydroxy-9-(hydroxymethyl)-1,6-dimethyl-11-oxo-4-((2-phenylhydrazono-)methyl)-11*H*-dibenzo[*b*,*e*][1,4]dioxepine-7-carboxylic acid. Isolated yield: 33%, white solid. ^1^H NMR (acetone-*d*_6_, 500 MHz) and JMOD (acetone-*d*_6_, 125 MHz): see Tables 1 and 2. HRESIMS *m*/*z* 463.1148 [M − H]^−^ (calcd for C_24_H_19_N_2_O_8_^−^*m*/*z* 463.1141).

(1b) (*E*)-4-((2-(4-bromophenyl)hydrazono)methyl)-3,8-dihydroxy-9-(hydroxymethyl)-1,6-dimethyl-11-oxo-11*H*-dibenzo[*b*,*e*][1,4]dioxepine-7-carboxylic acid. Isolated yield: 21%, white solid. ^1^H NMR (acetone-*d*_6_, 500 MHz) and JMOD (acetone-*d*_6_, 125 MHz): see Tables 1 and 2. HRESIMS *m*/*z* 543.0424 [M + H]^+^ (calcd for C_24_H_20_BrN_2_O_8_^+^*m*/*z* 543.0403).

(1c) 2,4,7,9-tetrabromo-3,8-dihydroxy-1,6-dimethyl-11*H*-dibenzo[*b*,*e*][1,4]dioxepin-11-one. Isolated yield: 34%, white solid. ^1^H NMR (acetone-*d*_6_, 500 MHz) and JMOD (acetone-*d*_6_, 125 MHz): see Tables 1 and 2. HRESIMS *m*/*z* 586.7004 [M − H]^−^ (calcd for C_15_H_7_Br_4_O_5_^−^*m*/*z* 586.6986).

(1d) (2,4,7-tribromo-3,8-dihydroxy-1,6-dimethyl-11-oxo-11*H*-dibenzo[*b*,*e*][1,4]dioxep-in-9-yl)methyl acetate. Isolated yield: 19%, white solid. ^1^H NMR (acetone-*d*_6_, 500 MHz) and JMOD (acetone-*d*_6_, 125 MHz): see Tables 1 and 2. HRESIMS *m*/*z* 576.8142 [M − H]^−^ (calcd for C_18_H_12_Br_3_O_7_^−^*m*/*z* 576.8133).

(1e) 6,6′-((4,6-dihydroxy-2-methyl-1,3-phenylene)bis(methylene))bis(4-formyl-3,7-dihydroxy-1,9-dimethyl-11-oxo-11*H*-dibenzo[*b*,*e*][1,4]dioxepine-8-carboxylic acid). Isolated yield: 31%, white solid. ^1^H NMR (DMSO-*d*_6_, 500 MHz) *δ* 10.53 (s, 2H, H-8), 6.50 (s, 2H, H-5), 6.06 (s, 1H, H-3′′), 3.77 (s, 4H, H-8′), 2.61 (s, 6H, H-9′), 2.01 (s, 3H, H-7′′), 1.95 (s, 3H, H-9); ^13^C NMR (DMSO-*d*_6_, 125 MHz) *δ* 192.4 (C-8), 170.4 (C-7′), 164.4 (C-4), 163.5 (C-7, C-2′), 161.8 (C-2), 154.9 (C-2′′, C-4′′), 151.8 (C-6), 144.0 (C-4′), 136.6 (C-6′′), 129.7 (C-5′), 118.5 (C-3′), 116.8 (C-5), 116.2 (C-1′′, C-5′′), 113.7 (C-1′), 113.4 (C-3), 111.1 (C-1), 102.5 (C-3′′), 20.8 (C-8′), 20.3 (C-9), 16.8 (C-7′′), 14.0 (C-9′) ppm. HRESIMS *m*/*z* 835.1519 [M − H]^−^ (calcd for C_43_H_31_O_18_^−^*m*/*z* 835.1511).

(6a) 9-bromo-1,4,10-trihydroxy-5,8-dimethyl-3,7-dioxo-3,7-dihydro-1*H*-benzo[6,7][1,4]-dioxepino[2,3-*e*]isobenzofuran-11-carbaldehyde. Isolated yield: 33%, white solid. ^1^H-NMR (DMSO-*d*_6_, 500 MHz) *δ* 10.58 (s, 1H, H-8), 2.29 (s, 3H, H-9), 2.17 (s, 3H, H-9′); ^13^C-NMR (DMSO-*d*_6_, 125 MHz) *δ* 104.8 (C-1), 157.7 (C-2), 100.7 (C-3), 165.3 (C-4), 115.1 (C-5), 142.4 (C-6), 161.0 (C-7), 189.8 (C-8), 22.2 (C-9), 115.1 (C-1′), 151.0 (C-2′), 124.0 (C-3′), 145.4 (C-4′), 141.2 (C-5′), 125.7 (C-6′), 11.4 (C-9′) ppm. HRESIMS *m*/*z* 448.9415 [M − H]^−^ (calcd for C_18_H_10_BrO_9_^−^*m*/*z* 448.9508).

### 3.4. Structural elucidation of the compounds

All isolated compounds have good solubility in acetone. 1D and 2D NMR spectra were acquired on a Bruker AVANCE III 500 MHz spectrometer in acetone-*d*_6_. Chemical shifts in ppm are referenced to the residual solvent signal (acetone-*d*_6_: *δ*_H_ = 2.05, *δ*_C_ = 29.8 ppm). The HRESIMS spectra were recorded using a MicrOTOF–Q mass spectrometer on an LC-Agilent 1100 LC-MSD Trap spectrometer. Silica gel 60 (0.040–0.063 mm, Himedia) was used for column chromatography. Analytical TLC was carried out on aluminum plates precoated with silica gel 60 F_254_ or silica gel 60 RP-18 F_254S_ (Merck), and eluted zones were visualized by spraying with 10% H_2_SO_4_ solution followed by heating.

### 3.5. Alpha-glucosidase inhibition assay

Evaluation of the inhibitory activity of compounds against yeast alpha-glucosidase followed a previous procedure.^30^

### 3.6. Inhibitory type assay of 1e on alpha-glucosidase

The mechanisms of inhibition of alpha-glucosidase by 1e were determined by Lineweaver–Burk plots using methods similar to those reported in the literature.^18^ Enzyme inhibition due to various concentrations of 1e was evaluated by monitoring the effects of different substrate concentrations. For Lineweaver–Burk double reciprocal plots 1/enzyme velocity (1/*V*) *vs.* 1/substrate concentration (1/[S]), the inhibition type was determined using various concentrations of *p*NPG (1 mM, 2 mM, and 4 mM) as a substrate in the presence of different concentrations of the test compounds (0, 1.82, 3.64, 7.28, and 14.57 μM for 1e). The experiments were carried out in 3 replicates. The mixtures were incubated at 37 °C and the optical density was measured at 405 nm every 1 minutes for 30 minutes with the ELx800 Absorbance Microplate Reader (BioTek Instruments, Inc., Vermont, USA). Optimal concentrations of the tested compounds were chosen based on the IC_50_ values. The inhibition constants were obtained graphically from secondary plots (Microsoft Excel 2010, Washington, USA).

### 3.7. Molecular docking

Molecular docking analyses were conducted using AutoDock Tools 1.5.7 and AutoDock Vina 1.1.2.^34^ The structural data for processing alpha-glucosidase I (PDB ID: 4 J5T) were obtained from the Protein Data Bank. The 2D visualization of 1e and its interaction with the protein was conducted using Poseview.

### 3.8. Molecular dynamics (MD) simulation

The docked conformation of the compound 1e binding to the 4j5t protein was employed to perform MD simulation based on GROMACS 5.1.3 with Amber99SB-ILDN force field.^35^ Antechamber software and ACPYNE python script^36^ were adopted to generate the topology and force field parameters of the ligand. To mimic the physiological ambience, the TIP3P water modeling^37^ was applied in a dodecahedron periodic boundary condition box, followed by water replacement by adequate sodium ions to generate a neutral solvated system. This system was then computed to energy minimization with 50 000 steps of steepest descent algorithm and thermodynamics equilibrium with the 100 ps-NVT (*T* = 300 K) and 100 ps-NPT (*T* = 300 K, *P* = 1 atm) ensembles. Finally, the 100 ns-MD simulation was produced at 2 fs temporal resolution. Afterward, the analysis of MD was performed using various packages embedded in the GROMACS and supplemented by XMGRACE software for visualization of extracted graphs. The binding free energy built on MD configurations was estimated by the molecular mechanics/Poisson–Boltzmann surface area (MM/PBSA) method,^38^ which consists of electrostatic and van der Waals interactions in the gas phase (Δ*E*_elec_, Δ*E*_vdW_, respectively), polar solvation energy (Δ*G*_polar_), and SASA-based non-polar solvation energy (Δ*G*_sa_) according to the following equation: Δ*E*_elec_ + Δ*E*_vdW_ + Δ*G*_polar_ + Δ*G*_sa_.

## 4. Conclusions

Through bioactivity-guided isolation focusing on alpha-glucosidase inhibition, eleven compounds were extracted and structurally characterized from the Vietnamese lichen *Usnea baileyi*, including protocetraric acid (1), 8′-methylstictic acid (2), stictic acid (3), 4,6-diformyl-8-hydroxy-3-methoxy-1,9-dimethyl-11-oxo-11*H*-dibenzo[b,e][1,4]dioxepine-7-carboxylic acid (4), vicanicin (5), norstictic acid (6), diffractaic acid (7), barbatic acid (8), atranol (9), 5-chlorohaematommic acid (10), and eumitrin A1 (11). From 1 and 6, new derivatives 1a–1e and 6a were synthesized. Compounds 1–6, 1a–1e, 6a, and 11 exhibited significant alpha-glucosidase inhibition, with IC_50_ values ranging from 10.4 to 130 μM. A kinetic study revealed that 1e acts as a mixed inhibitor. Molecular docking was applied to potent compounds (1–3, 6, 1a–1e, and 6a) to clarify the inhibitory mechanism. Compounds 1–6, 1a–1e, and 6a showed non cytotoxicity against a normal cell line. These findings contribute to the known chemical diversity of the lichen *Usnea baileyi* for further studies on the antidiabetic potential of this valuable source. Future studies could investigate the potent synergistic effects of the reported compounds in combination.

## Data availability statement

The data supporting this article have been included as part of the ESI.†

## Conflicts of interest

No potential conflict of interest was reported by the authors.

## Supplementary Material

RA-014-D4RA04449E-s001
