# Supplementary material for: Alpha-glucosidase inhibitory compounds from Vietnamese lichen Usnea baileyi: in vitro and in silico aspects
Source: RSC Adv. 2024 Oct 15;14(44):32624–36. doi: 10.1039/d4ra04449e (PMC11475664; doi:10.1039/d4ra04449e)
Supplement: RA-014-D4RA04449E-s001 [file RA-014-D4RA04449E-s001.pdf]

This journal is © The Royal Society of Chemistry 2024

SUPPORTING INFORMATION

Alpha-Glucosidase Inhibitory Compounds from Vietnamese Lichen *Usnea baileyi*: *in vitro* and *in silico* aspects

TABLE OF CONTENTS

Figure S1. Transformation **1** to **1e**

Figure S2-A. HRESIMS spectrum of **1a**

Figure S2-B. ^{1}H NMR (acetone-d_6, 500 MHz) spectrum of **1a**

Figure S2-C. ^{13}C-NMR (acetone-d_6, 125 MHz) spectrum of **1a**

Figure S2-D. HMBC (acetone-d_6) spectrum of **1a**

Figure S3-A. HRESIMS spectrum of **1b**

Figure S3-B. ^{1}H-NMR (acetone-d_6, 500 MHz) spectrum of **1b**

Figure S3-C. ^{13}C-NMR (acetone-d_6, 125 MHz) spectrum of **1b**

Figure S3-D. HSQC (acetone-d_6) spectrum of **1b**

Figure S4-A. HRESIMS spectrum of **1c**

Figure S4-B. ^{1}H-NMR (acetone-d_6, 500 MHz) spectrum of **1c**

Figure S4-C. JMOD (acetone-d_6, 125 MHz) spectrum of **1c**

Figure S5-A. HRESIMS spectrum of **1d**

Figure S5-B. ^{1}H-NMR (acetone-d_6, 500 MHz) spectrum of **1d**

Figure S5-C. JMOD and ^{13}C spectra (acetone-d_6, 125 MHz) of **1d**

Figure S6-A. HRESIMS spectrum of **1e**

Figure S6-B. ^{1}H NMR (DMSO-d_6, 500 MHz) spectrum of **1e**

Figure S6-C. ^{13}C NMR (DMSO-d_6, 125 MHz) spectrum of **1e**

Figure S6-D. HSQC (DMSO-d_6) spectrum of **1e**

Figure S6-E. HMBC (DMSO-d_6) spectrum of **1e**

Figure S7-A. HRESIMS spectrum of **6a**

Figure S7-B. ^{1}H NMR (acetone-d_6, 500 MHz) spectrum of **6a**

Figure S7-C. JMOD (acetone-d_6, 125 MHz) spectrum of **6a**

Figure S8. Transformation **1** to **1e**

Table S1. Alpha-glucosidase inhibitory activity of the crude extracts and the fractions prepared from crude EtOAc extract.

Table S2. Cytotoxicity of compounds **1-6, 11, 1a-1e**, and **6a**

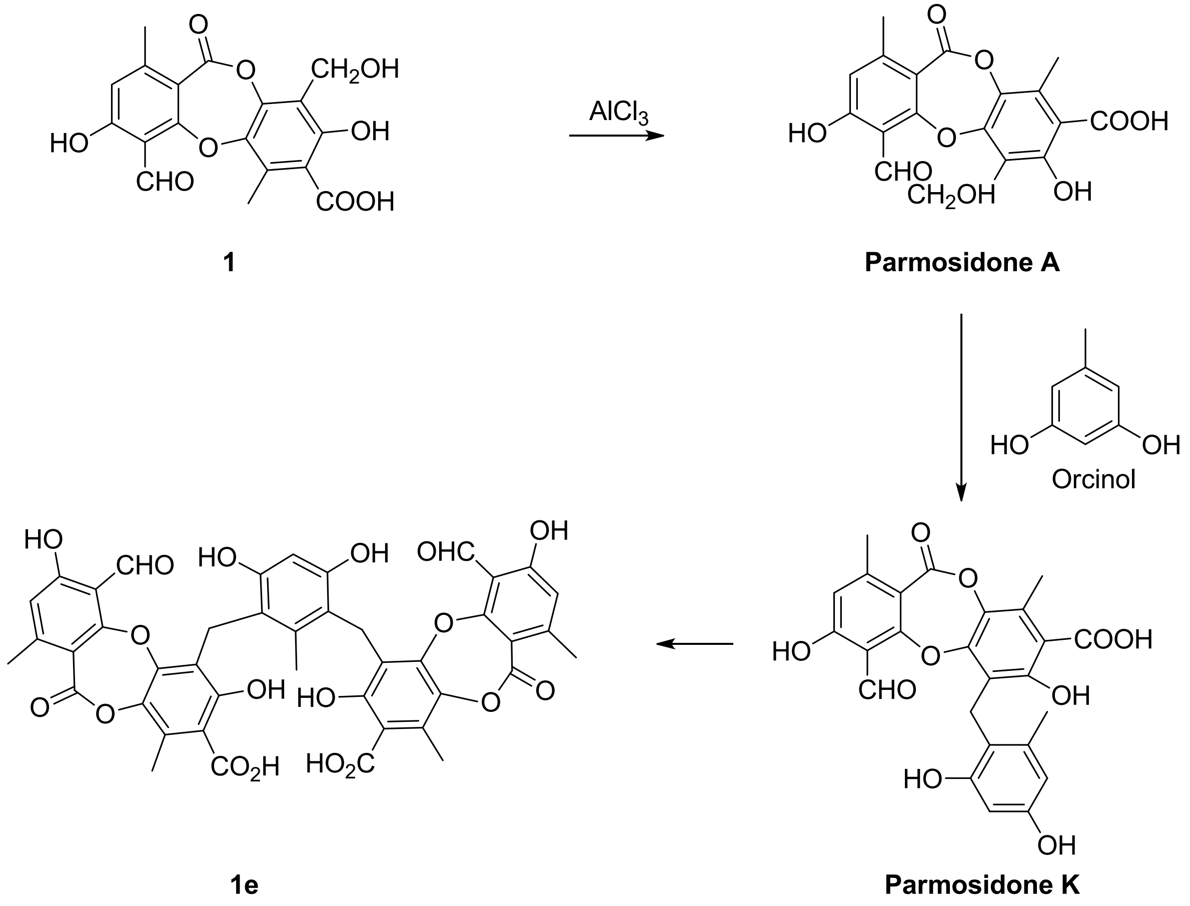

Figure S1. Transformation **1** to **1e**

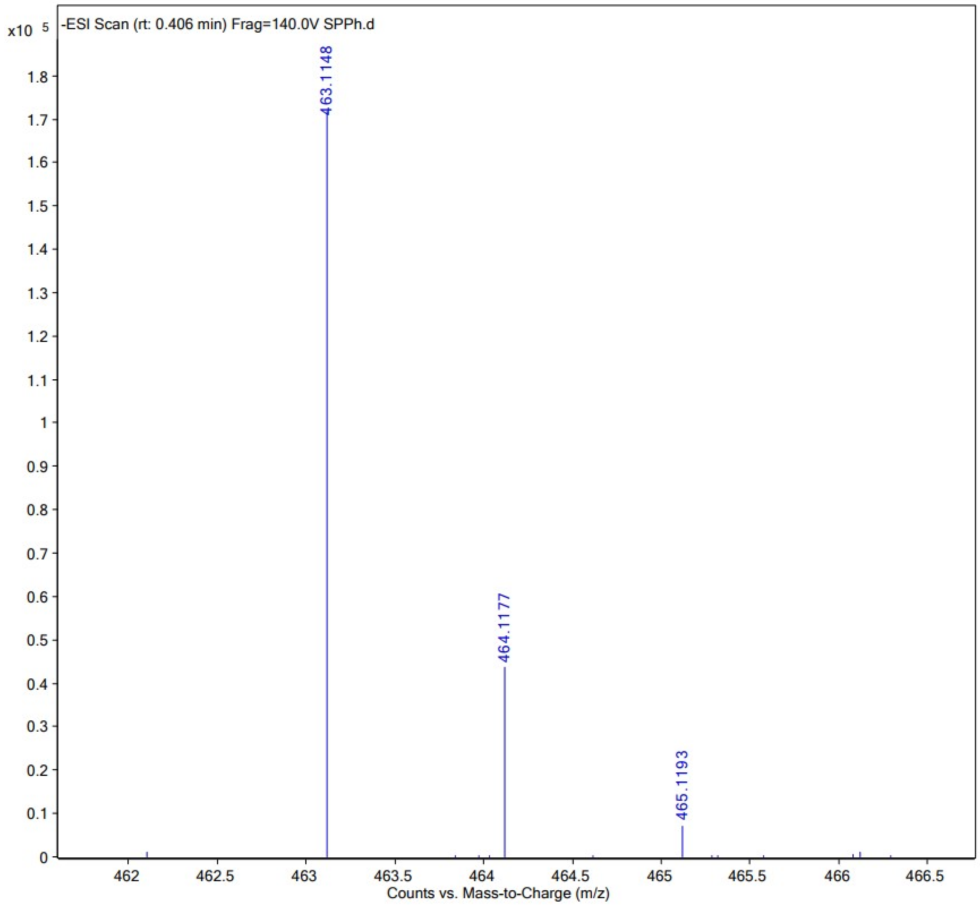

Figure S2-A. HRESIMS spectrum of **1a**

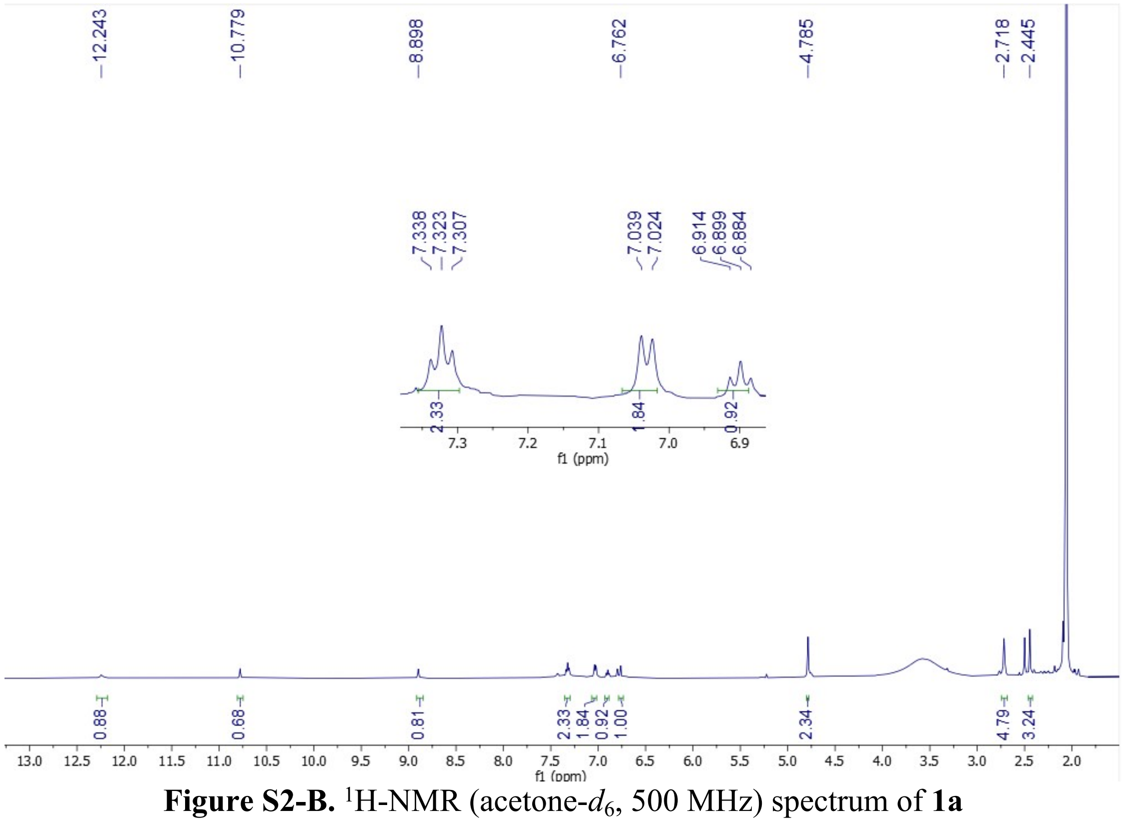

Figure S2-B. ^{1}H-NMR (acetone-d_6, 500 MHz) spectrum of **1a**

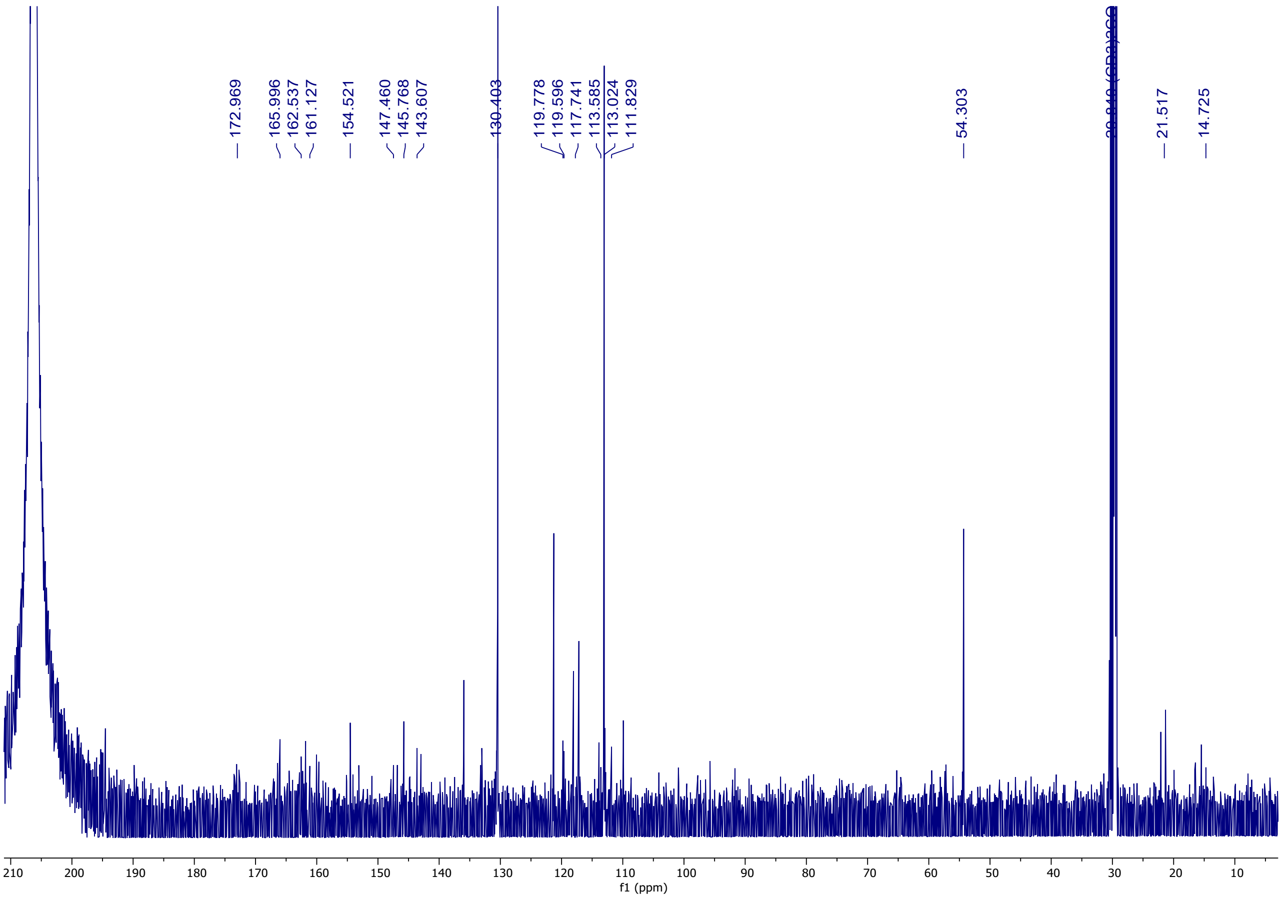

Figure S2-C. ^{13}C-NMR (acetone-d_6, 125 MHz) spectrum of **1a**

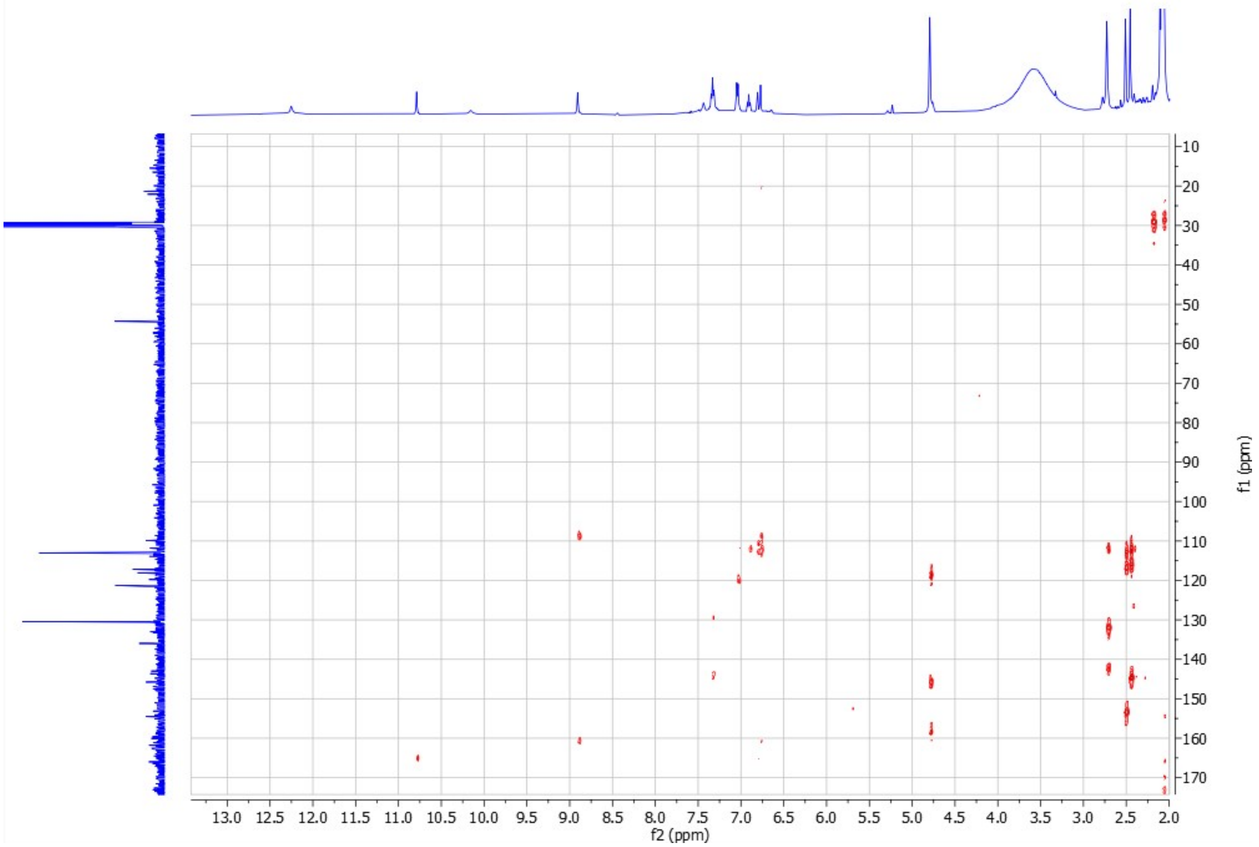

Figure S2-D. HMBC (acetone-d_6) spectrum of **1a**

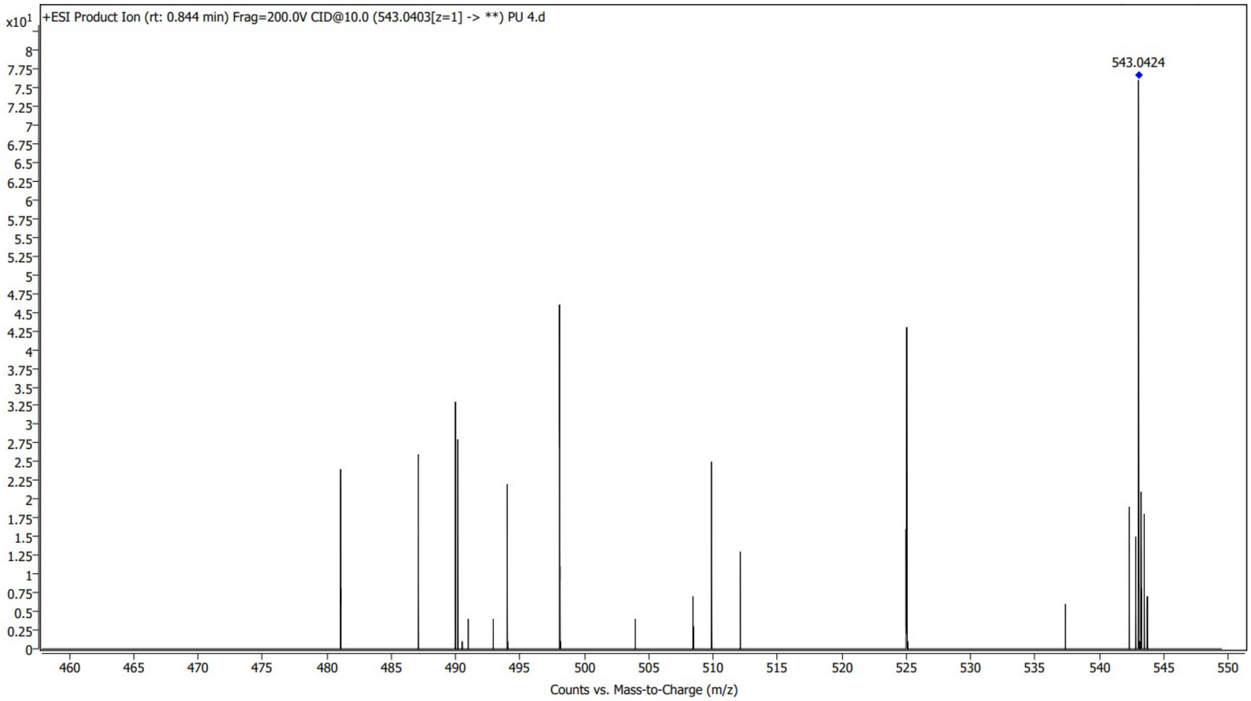

Figure S3-A. HRESIMS spectrum of **1b**

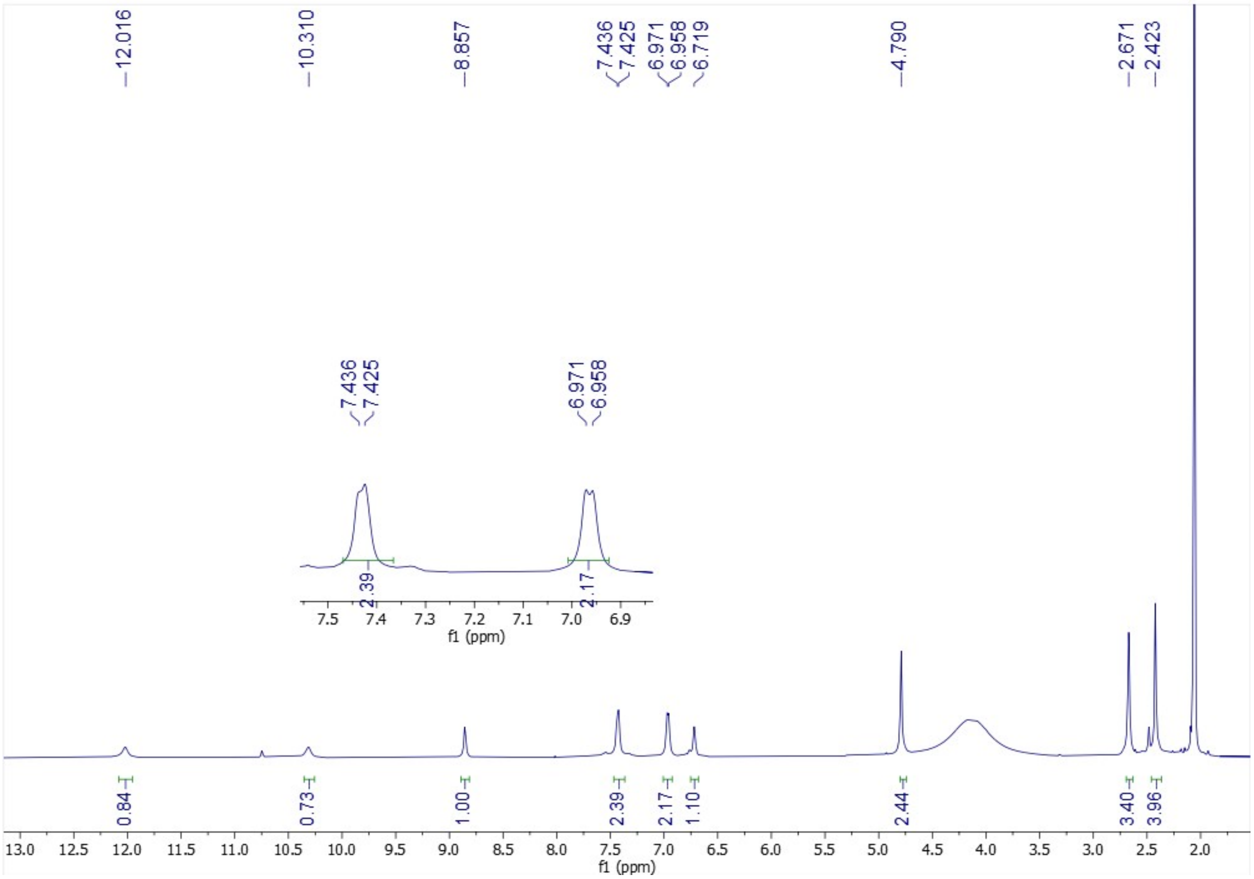

Figure S3-B. ^{1}H-NMR (acetone-d_6, 500 MHz) spectrum of **1b**

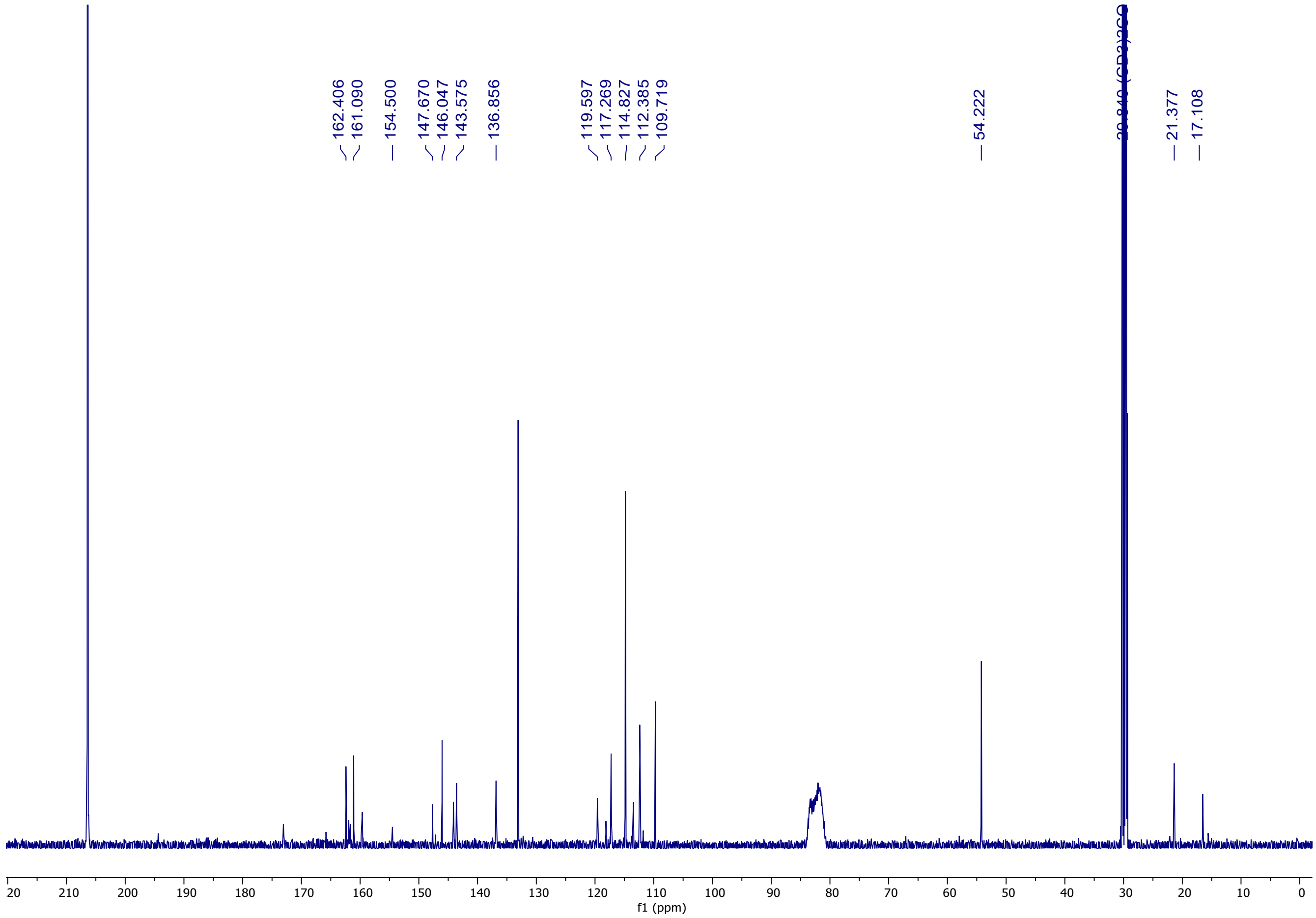

Figure S3-C. ^{13}C-NMR (acetone-d_6, 125 MHz) spectrum of **1b**

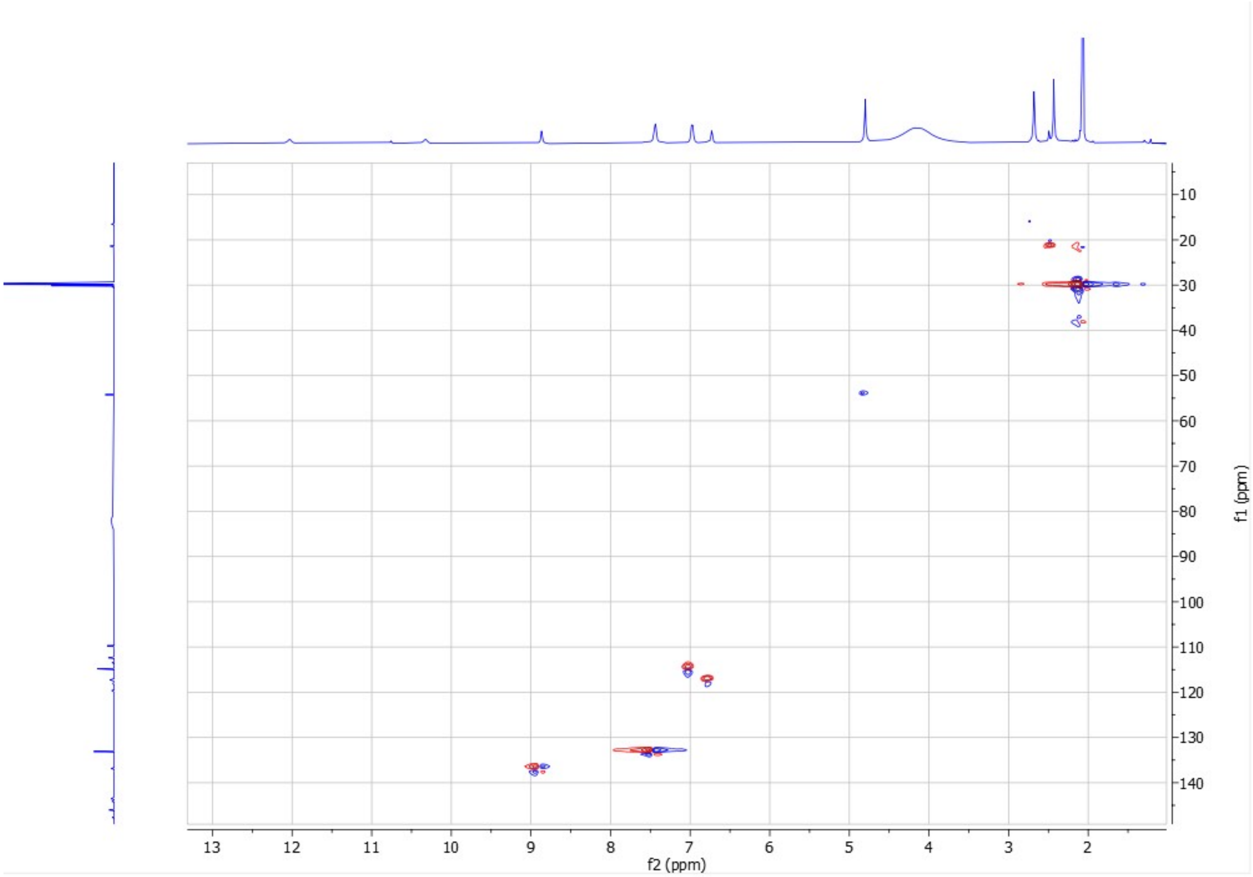

Figure S3-D. HSQC (acetone-d_6) spectrum of **1b**

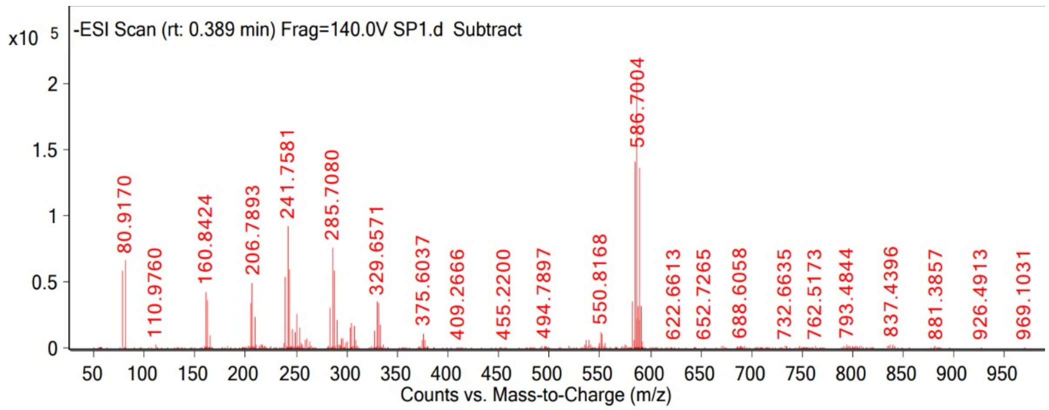

Figure S4-A. HRESIMS spectrum of **1c**

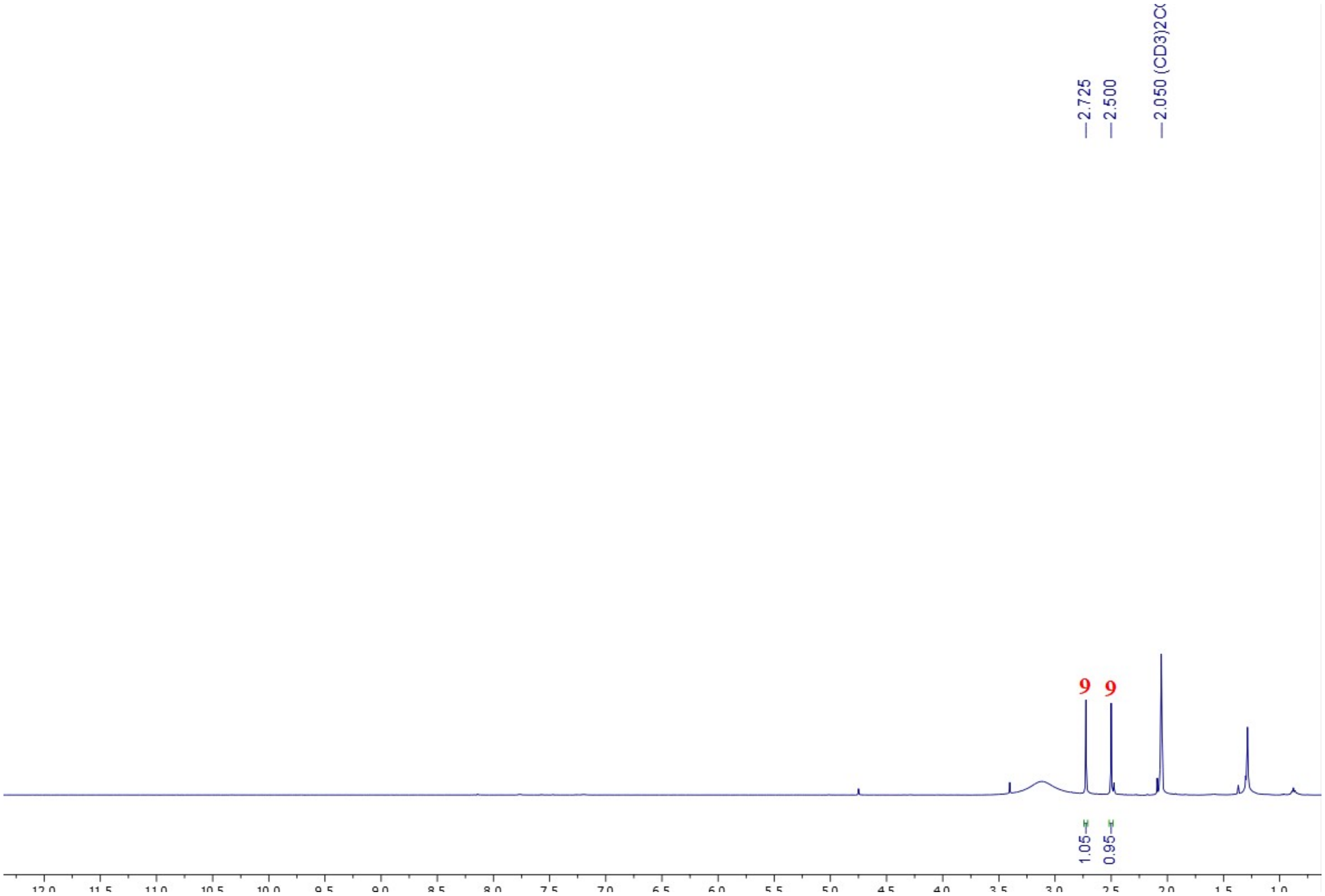

Figure S4-B. ^{1}H-NMR (acetone-d_6, 500 MHz) spectrum of **1c**

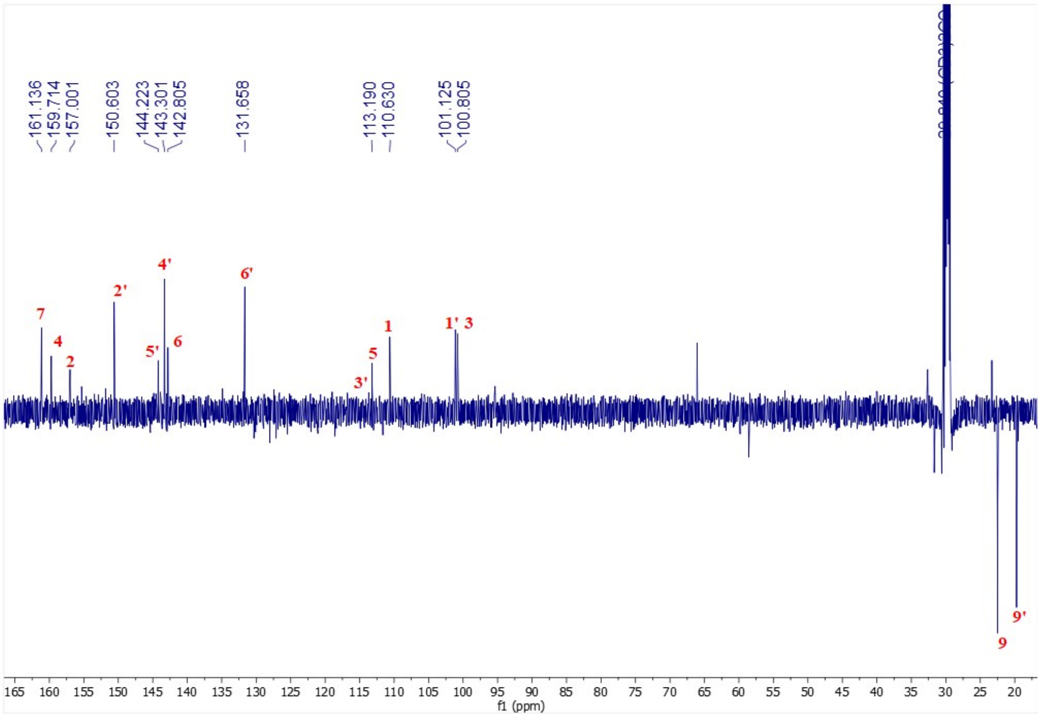

Figure S4-C. JMOD (acetone-d_6, 125 MHz) spectrum of **1c**

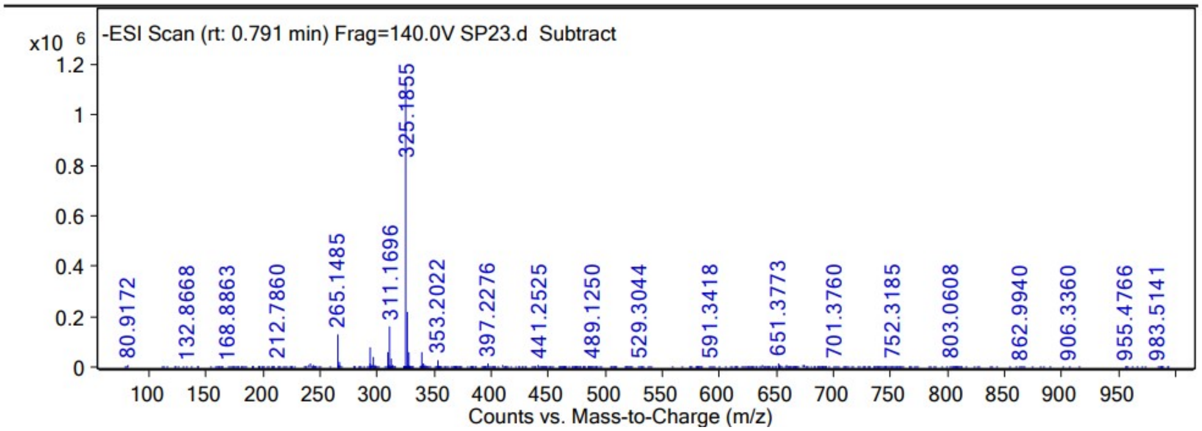

573.2203	2	244.77
573.9122		203.06
574.7172	1	1782.84
574.9332		386.34
574.9924		412.2
575.0315		224.32
575.7163	1	237.88
575.7721		207.83
576.7159	1	2087.6
576.8142	1	20902.08
576.9439		352.38
577.0114		286.19
577.0541		386.07
577.2051		211.16
577.3543		340.89
577.4006		334.22

577.6627		241.2
577.7104	1	503.45
577.8174	1	4372.84
578.0976		263.15
578.714	1	1586.43
578.8127	1	62467.27
579.3608		412.12
579.4896		219.86
579.6746		280.21
579.7105	1	291.28
579.8162	1	11807.33
579.9693		272.47
580.7102	1	544.9
580.811	1	64009.91
581.2263		257.39

Figure S5-A. HRESIMS spectrum of **1d**

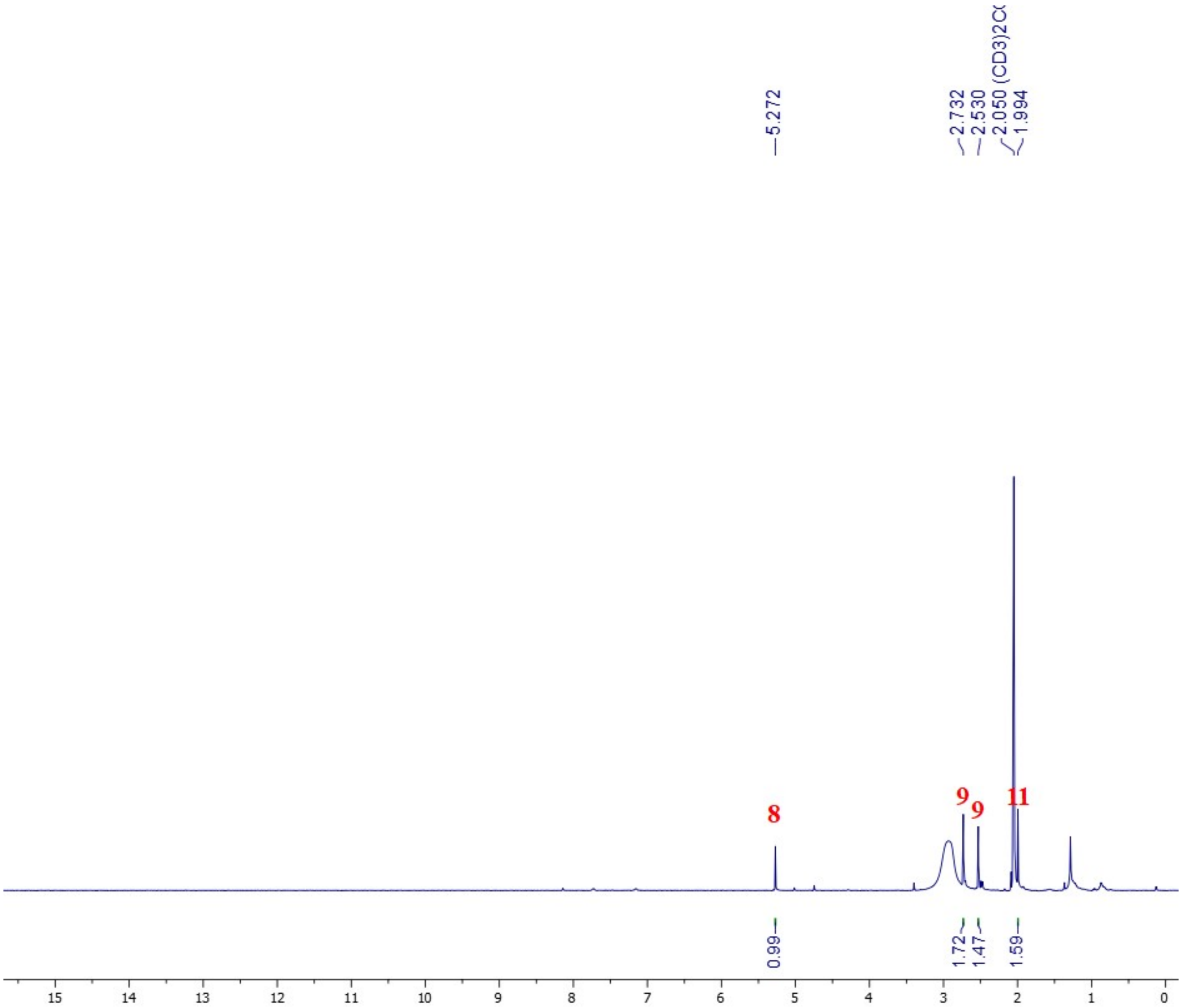

Figure S5-B. ^{1}H-NMR (acetone-d_6, 500 MHz) spectrum of **1d**

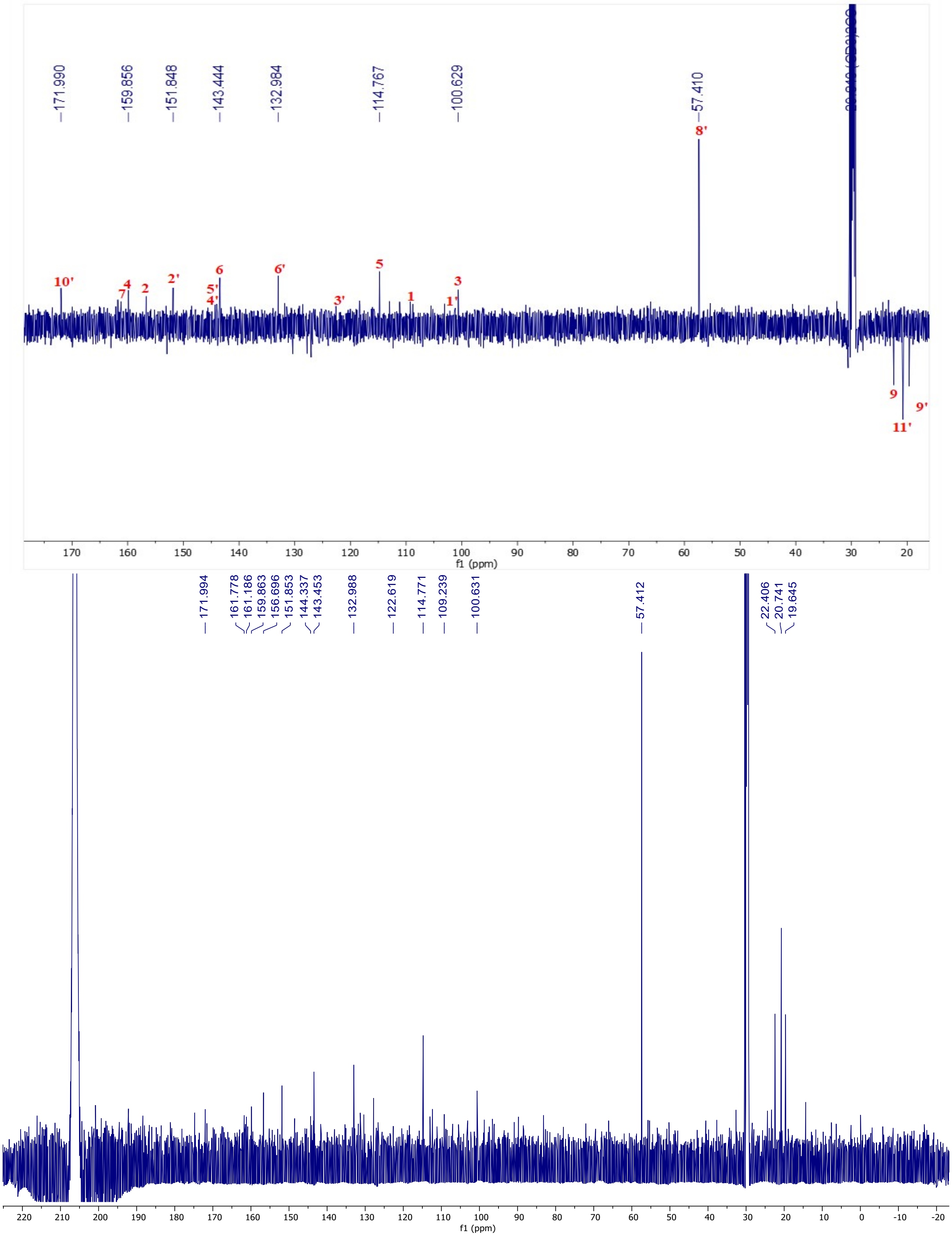

Figure S5-C. JMOD and ^{13}C spectra (acetone-d_6, 125 MHz) of **1d**

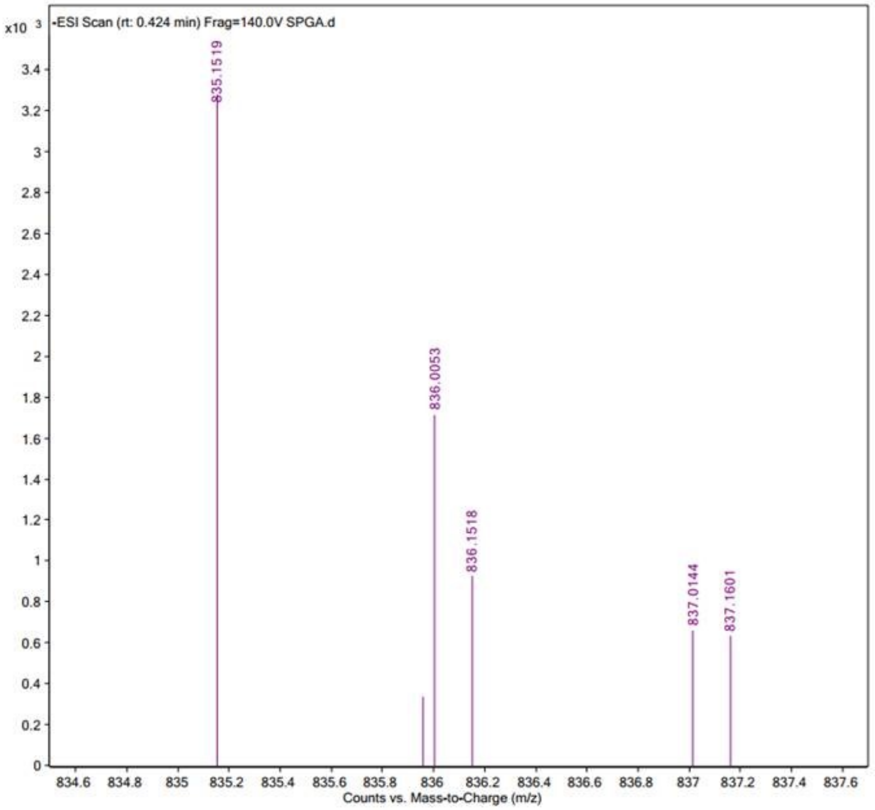

Figure S6-A. HRESIMS spectrum of **1e**

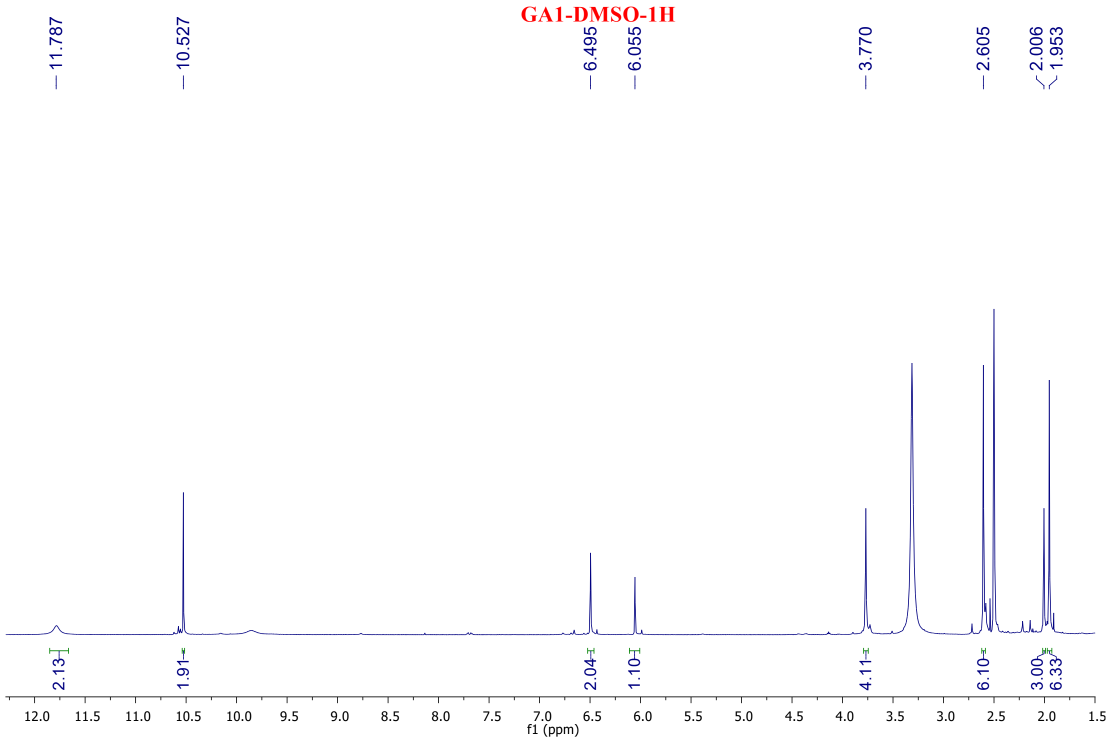

Figure S6-B. ^{1}H NMR (DMSO-d_6, 500 MHz) spectrum of **1e**

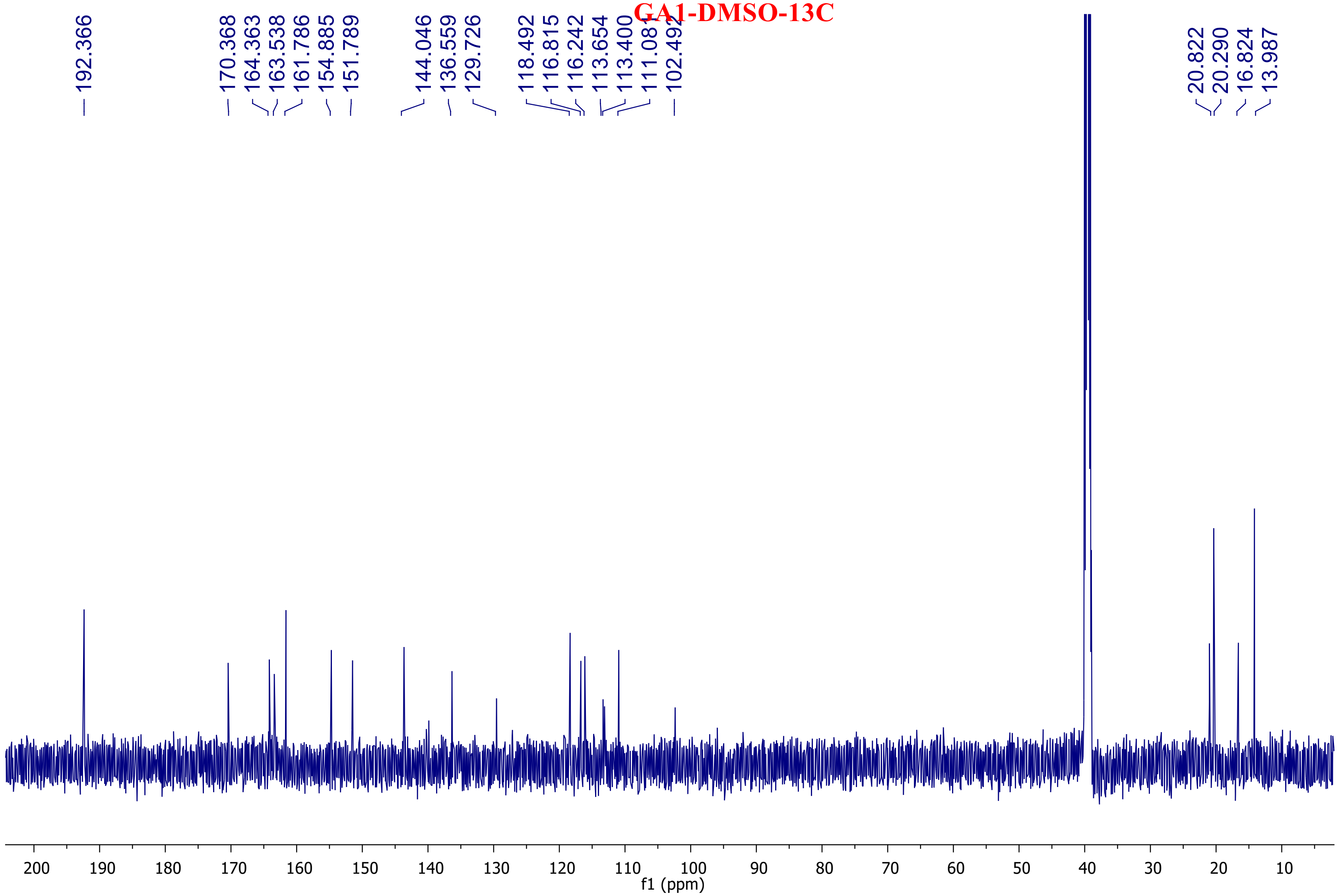

Figure S6-C. ^{13}C NMR (DMSO-d_6, 125 MHz) spectrum of **1e**

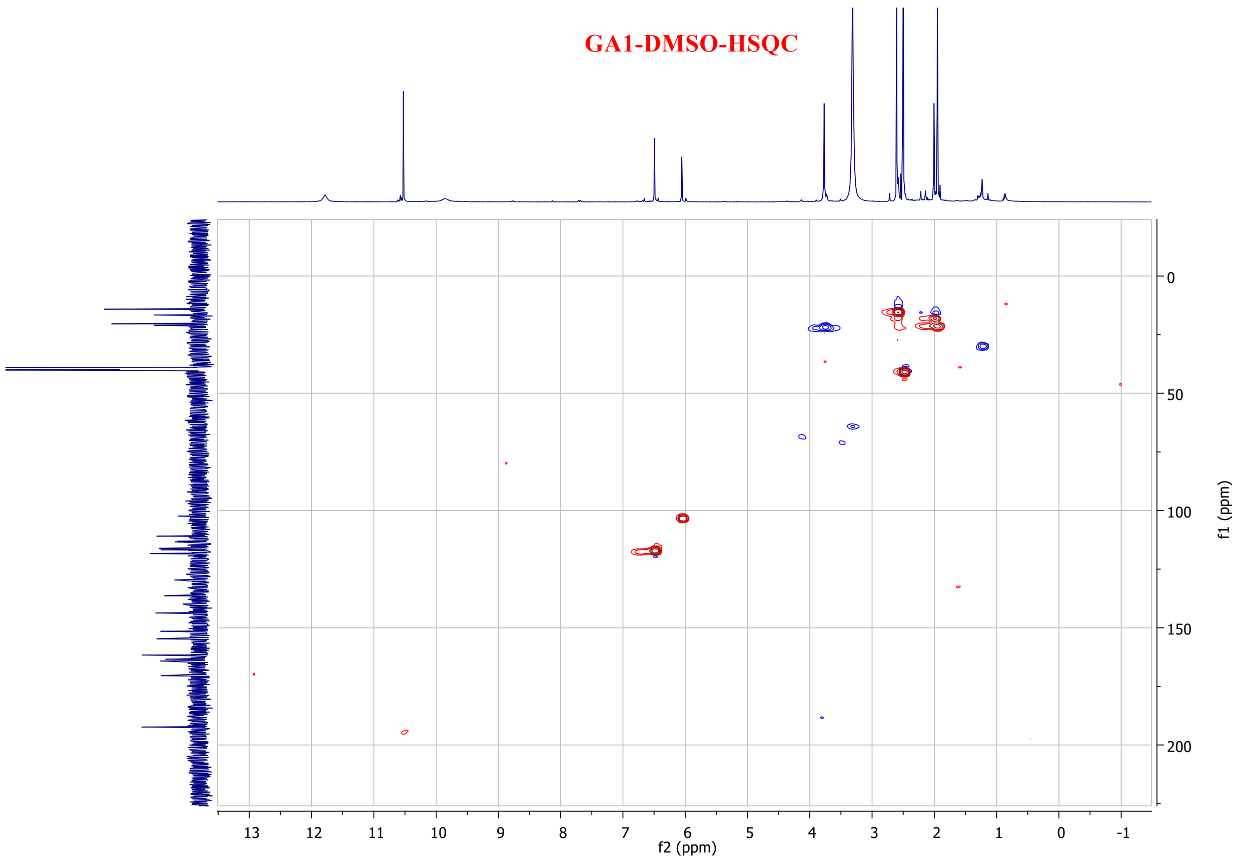

Figure S6-D. HSQC (DMSO-d_6) spectrum of **1e**

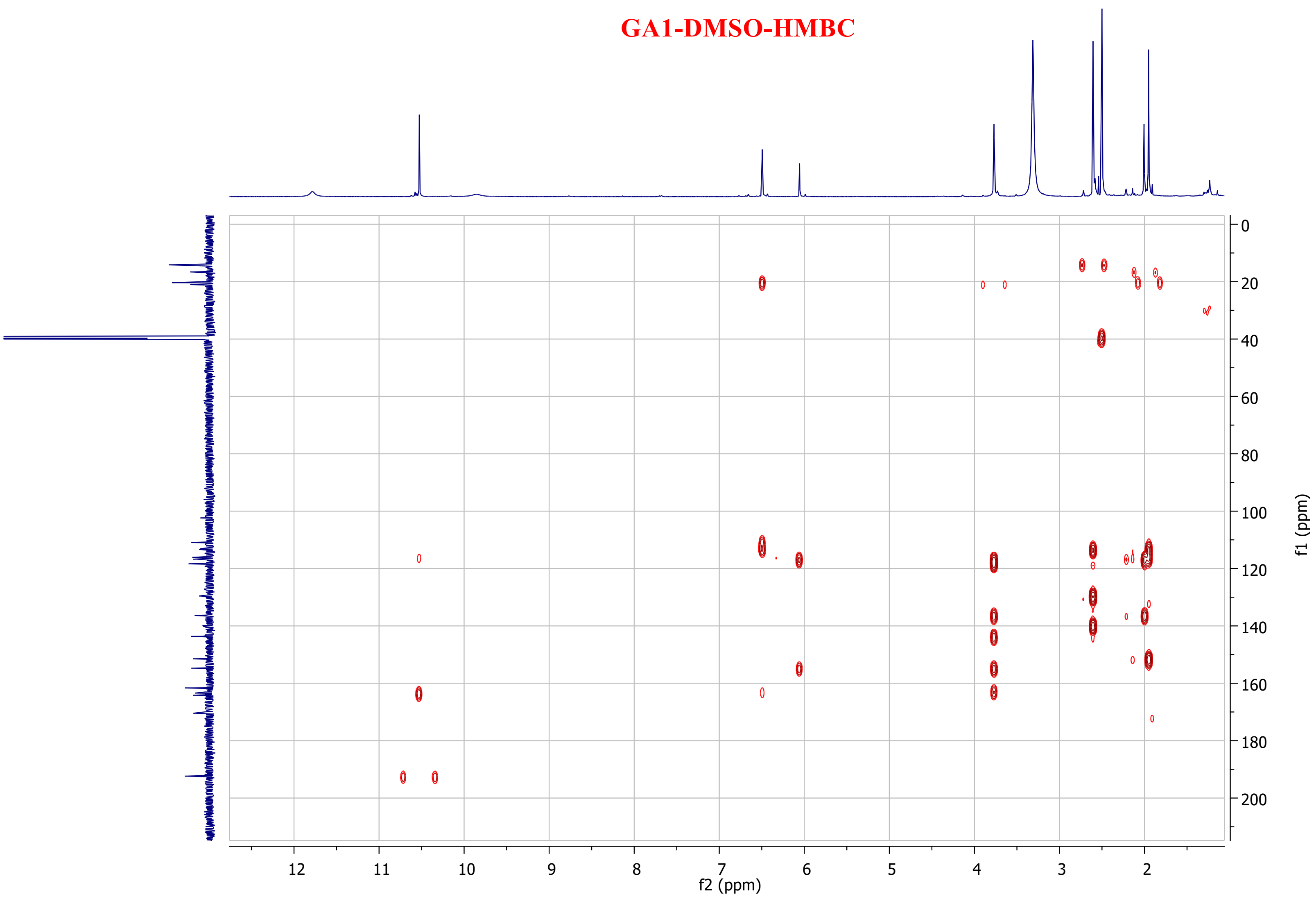

Figure S6-E. HMBC (DMSO-d_6) spectrum of **1e**

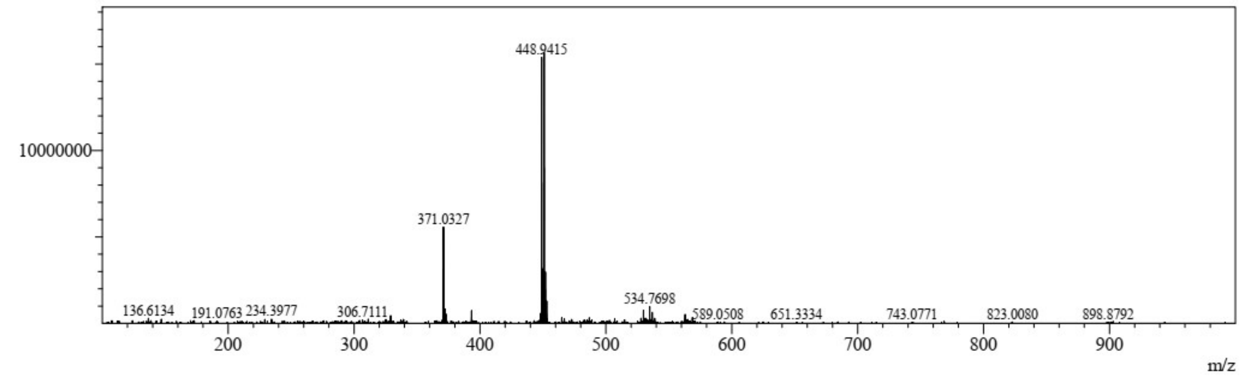

Figure S7-A. HRESIMS spectrum of **6a**

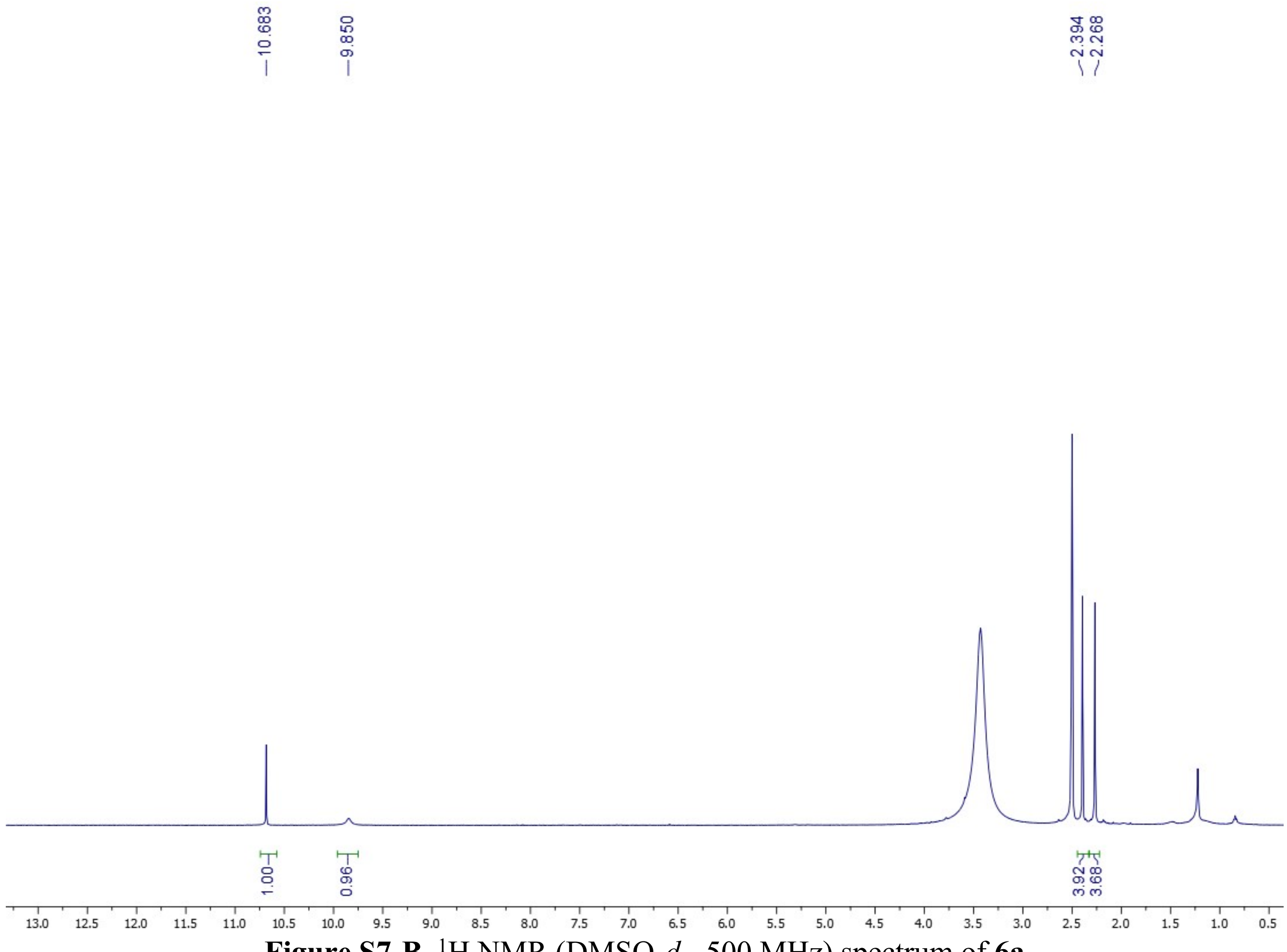

Figure S7-B. ^{1}H NMR (DMSO-d_6, 500 MHz) spectrum of **6a**

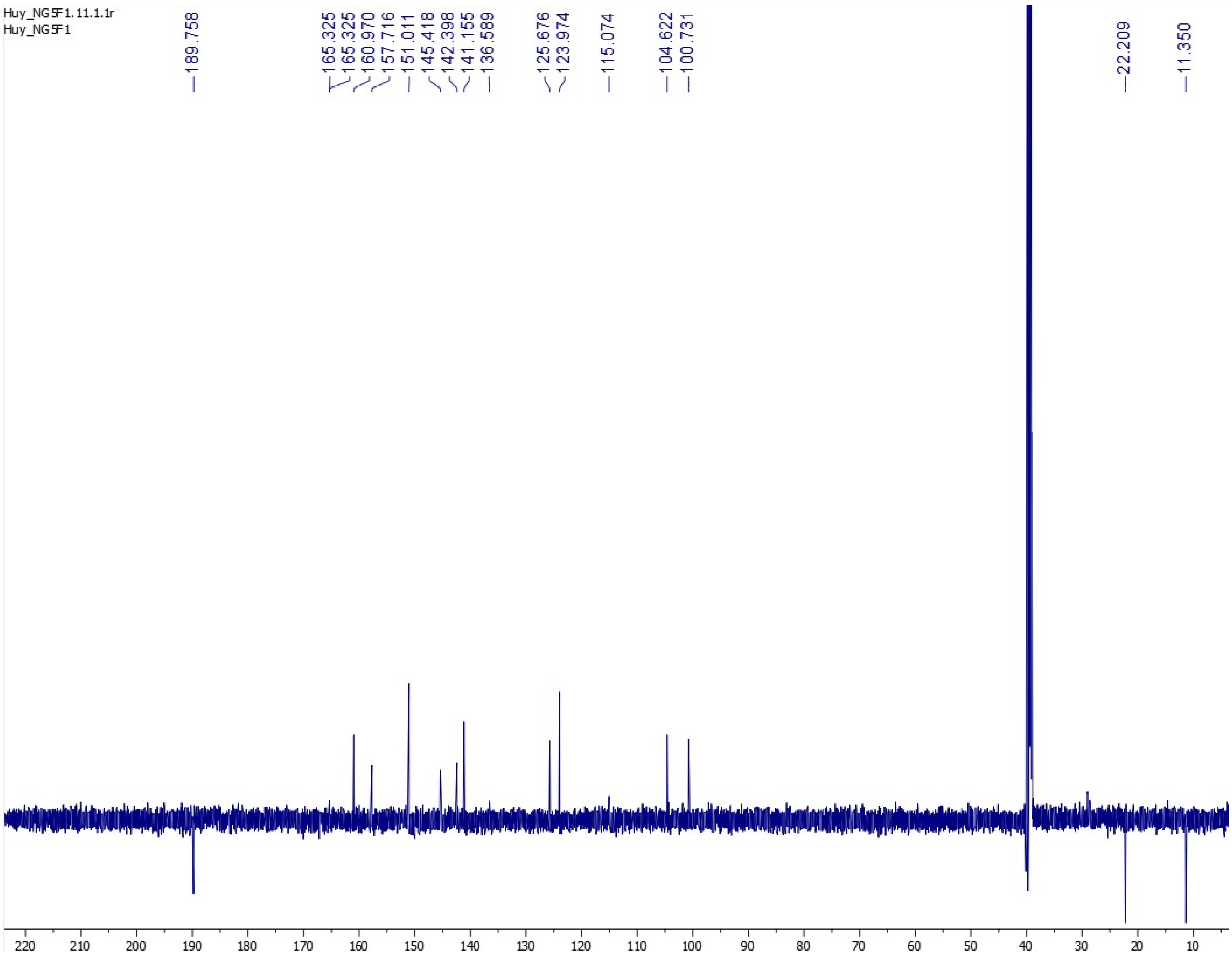

Figure S7-C. JMOD (DMSO-d_6, 125 MHz) spectrum of **6a**

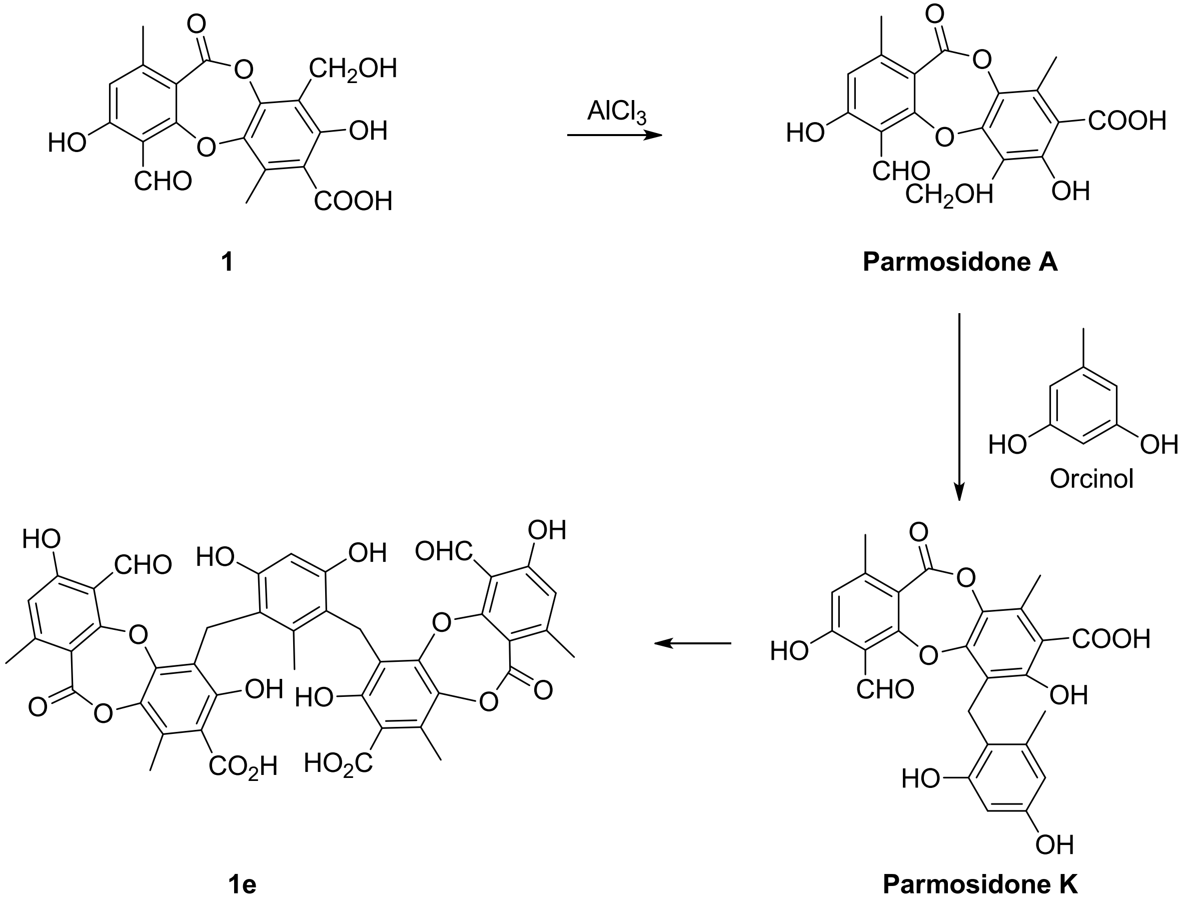

Figure S8. Transformation **1** to **1e**

Table S1. Alpha-glucosidase inhibitory activity of the crude extracts and the fractions prepared from crude EtOAc extract.

**Sample**	**IC_{50}** (μg/mL)
Crude *n*-hexane extract	>200
Crude EtOAc extract	34.5 ± 1.2
Crude MeOH extract	96.1 ± 5.8
Fr. DY1	>200
Fr. DY2	>200
Fr. DY3	45.6 ± 4.6
Fr. DY4	33.3 ± 1.2
Fr. DY5	15.1 ± 1.3
Fr. DY6	24.3 ± 0.7
Fr. DY7	>200
Fr. DY8	>200
Fr. DY9	>200
Fr. DY10	>200

Table S2. Cytotoxicity of compounds **1-6**, **11**, **1a-1e**, and **6a**

**Ligand**	**Hek293** **IC_{50} (µg/mL)**	**HepG2** **IC_{50} (µg/mL)**
**1**	35.73 ± 2.4	63.6 ± 0.06
**1a**	>100	>100
**1b**	>100	>100
**1c**	58.89 ± 2.84	76.51 ± 1.07
**1d**	61.13 ± 2.71	86.7 ± 0.68
**1e**	>100	>100
**2**	>100	>100
**3**	33.9 ± 1.6	>100
**4**	>100	>100
**5**	>100	>100
**6**	>100	>100
**6a**	88.15 ± 2.02	>100
**11**	37.62 ± 4.3	38.8 ± 0.5
Doxorubicin	2.13 ± 0.01	1.89 ± 0.03

Cytotoxicity Assay

The method used followed that reported by Nguyen et al. (2022). Briefly, HepG2 and Hek293 cells in a complete medium containing DMEM (HyClone, Cytiva, USA) and 10% fetal bovine serum (FBS) were seeded into a 96-well plate at 5×10^4 cells per well. The cells were then incubated at 37°C with 5% CO_2. After culturing for 24 h, the old medium was replaced by diluted compounds (in DMSO), positive control Doxorubicin (DOX, Fesenius Kabi, Germany), and negative control dimethyl sulfoxide (DMSO, Sigma-Aldrich, Germany). After 72 h of treatment, MTT (3-(4,5-Dimethyl-2-thiazolyl)-2,5-diphenyl-2H-tetrazolium bromide) was added into each well of the test plate. The plate was incubated at 37°C with 5% CO_2 for another 3 h. DMSO was used to dissolve the crystals from the interaction between MTT and viable cells. The absorbance of samples was read at 570 nm by the ELISA Reader (BioTek, USA). Cell death (% inhibition) was estimated by the following formula:

$$\% \text{ Inhibition} = 100 - [100 \times (A_{Sample} - A_{B1})/(A_{DMSO} - A_{B2})]$$

Where, A_{Sample}: Absorbance of sample, A_{DMSO}: Absorbance of negative control, A_{B1}: Absorbance of blank of sample, A_{B2}: Absorbance of blank of DMSO. The IC_{50} value and the inhibitory chart were respectively calculated and built using GraphPad Prism software (version 8.0.1, Insightful Science LLC, USA).

Ref: Nguyen HH, Aree T, Nguyen HT, et al. Diorygmones A-B, two new guaiane-sesquiterpenes from the cultured lichen mycobiont of *Diorygma sp. Natural Product Research*. Published online February 1, 2023:1-6. doi:10.1080/14786419.2023.2172007
